# Screening of candidate G-quadruplex ligands for the human *c-KIT* promotorial region and their effects in multiple *in-vitro* models

**DOI:** 10.18632/oncotarget.7808

**Published:** 2016-03-01

**Authors:** Eleonora Zorzan, Silvia Da Ros, Caterina Musetti, Lara Zorro Shahidian, Nuno Filipe Ramos Coelho, Federico Bonsembiante, Sébastien Létard, Maria Elena Gelain, Manlio Palumbo, Patrice Dubreuil, Mery Giantin, Claudia Sissi, Mauro Dacasto

**Affiliations:** ^1^ Department of Comparative Biomedicine and Food Science, University of Padua, Legnaro, Padua, Italy; ^2^ Department of Pharmaceutical and Pharmacological Sciences, University of Padua, Padua, Italy; ^3^ Centre de Recherche en Cancerologie de Marseille, INSERM (U1068), CNRS (U7258), Université Aix-Marseille (UM105), Marseille, France

**Keywords:** *c-KIT*, G-quadruplex, *in-vitro* models, G4-ligands, anthraquinone

## Abstract

Stabilization of G-quadruplex (G4) structures in promoters is a novel promising strategy to regulate gene expression at transcriptional and translational levels. *c-KIT* proto-oncogene encodes for a tyrosine kinase receptor. It is involved in several physiological processes, but it is also dysregulated in many diseases, including cancer. Two G-rich sequences able to fold into G4, have been identified in *c-KIT* proximal promoter, thus representing suitable targets for anticancer intervention. Herein, we screened an “in house” library of compounds for the recognition of these G4 elements and we identified three promising ligands. Their G4-binding properties were analyzed and related to their antiproliferative, transcriptional and post-transcriptional effects in MCF7 and HGC27 cell lines. Besides *c-KIT*, the transcriptional analysis covered a panel of oncogenes known to possess G4 in their promoters.

From these studies, an anthraquinone derivative (AQ1) was found to efficiently downregulate *c-KIT* mRNA and protein in both cell lines. The targeted activity of AQ1 was confirmed using *c-KIT*–dependent cell lines that present either *c-KIT* mutations or promoter engineered (i.e., α155, HMC1.2 and ROSA cells).

Present results indicate AQ1 as a promising compound for the target therapy of c-*KIT*-dependent tumors, worth of further and in depth molecular investigations.

## INTRODUCTION

The *c-KIT* proto-oncogene (*c-KIT*) codes for a tyrosine kinase receptor (c-kit) that, once activated by stem cell factor (SCF) in mast cells, melanocytes and Cajal interstitial cells, participates in a broad range of physiological processes, including cell proliferation, migration, maturation and survival [1, 2].

*c-KIT* is dysregulated in many diseases, including cancer [3]; in neoplastic diseases, its increased expression and auto-phosphorylation allows tumor cells to develop independently from growth and survival signals [4, 5]. Furthermore, several mutations potentially leading to c-kit activation in the absence of SCF binding have been reported [6]. Gain of function mutations can be found in gastrointestinal stromal tumor (GIST, >90%), mast cell tumors (>70%), nasal T-cell lymphomas (>17%), seminoma/dysgerminoma (>9%) and some acute myeloid leukemia (>68%) [7].

Less than fifteen years ago, tyrosine kinase inhibitors (TKIs) were approved for the treatment of human cancers overexpressing c-kit. The immediate results obtained using TKIs were promising, but drug-resistance phenomena were soon observed for some of them, e.g. imatinib [8] as a result of several cellular mechanisms. Moreover, the same drug can show differential clinical responses depending on the presence of a wild type or a mutated *c-KIT* genotype [9]. This highlighted the need of novel pharmacological tools to block c-kit activity.

Recently, within the human *c-KIT* promoter, two guanine-rich sequences have been identified, i.e. KIT1 and KIT2, occurring respectively between positions −12 and −34 bp and positions −64 and −84 bp upstream the transcription starting site [10-12]. These sequences have been confirmed to fold into non-canonical structures named G-quadruplex (G4), formed by stacked G-tetrads, each constituted by four guanines connected through a Hoogsteen-hydrogen bonds network to provide a square planar platform [13]. G4 structures have been shown to act as regulatory elements making them a potentially attractive target to be exploited for the regulation of gene expression at transcriptional level [14-18]. Currently, several small molecules that efficiently bind the G4 structures of *c-KIT* have been identified and most of them present an extended aromatic core that allows the stacking on the terminal G-tetrads [17,19]. For some of these ligands the inhibition of *c-KIT* expression has been confirmed in cells: these include trisubstituted isoalloxazines, naphthalene diimide derivatives, substituted indenoisoquinolines and benzo[a]phenoxazines [12, 20-22].

To further optimize the promising outcome provided by these derivatives, we set up a library of “in house” available compounds that can be clustered into five different families according to their main scaffold: anthraquinone (AQ) [23], anthracene (AN) [24], phenantroline (Phen) [25-27], naphthalene diimide (NDI) [28] and heterocyclic diamidines (HAD) [29]. Interestingly, G4 recognition properties were previously reported for at least one member of each family. On a comparative basis, most of structural variations concern the compound side chains, either in terms of composition or relative localization on the pharmacophore. This was a precise choice: in fact, upon stacking of the planar core, the side chains are available to achieve the selective recognition of G4 loops and grooves, which are the structural domains largely defining the unique conformational signature of G4s. According to this model, compounds able to drive the preferential recognition of nucleic acid structures which are structurally divergent in these portions, might be expected to modulate the affinity/selectivity towards different G4 arrangements.

In the present study, the whole library has been screened against the two G-rich sequences of *c-KIT*, to identify the most promising candidates to suppress *c-KIT* expression by the efficient stabilization of KIT1 and/or KIT2 G4 structures. Following the binding studies, three G4-ligands were selected and subsequently tested for cytotoxicity. Finally, their effects on *c-KIT* mRNA and protein expression were evaluated in a panel of human cancer cell lines, including also some well-known *in vitro* models of *c-KIT*-dependent tumors.

## RESULTS

### Ligands selection

**Figure 1:**
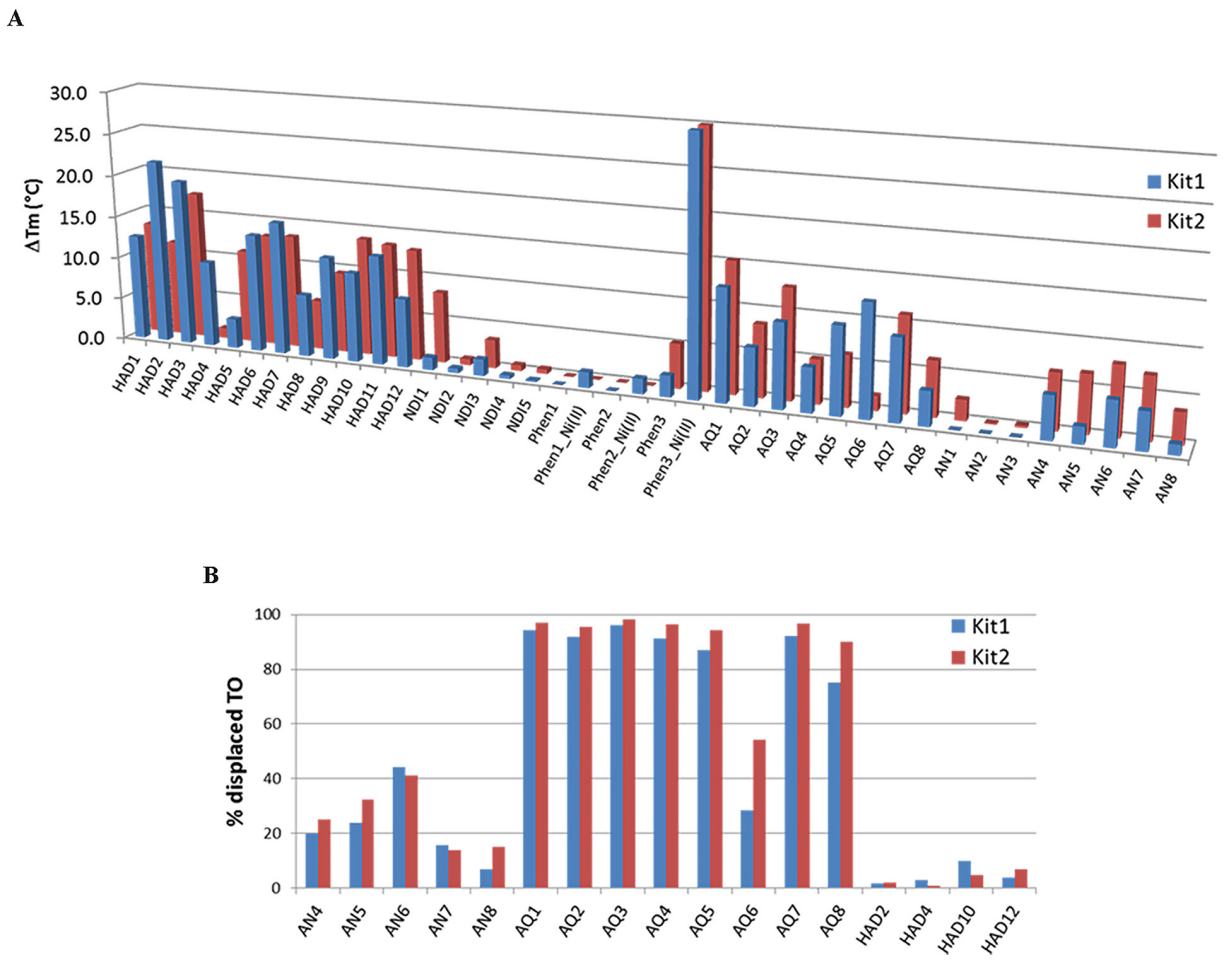
**A.** Increments of the melting temperature of the G4 arrangements of tested *c-KIT* sequences induced by 1 μM of tested ligands. Data were acquired in LiP buffer containing either 50 mM or 1 mM KCl for KIT1 or KIT2, respectively. **B.** Percentage of TO displacement from KIT1 or KIT2 induced by a 4-fold excess of AQ, AN and HAD derivatives.

All the members of our library were previously tested for their ability to stabilize the G4 structure of the human telomeric sequence as well as of a random DNA double helix, and a general preference for G4 *vs* dsDNA was observed for most of them [23-29]. Consistently, as a first preliminary screening tool we analyzed all the members of our library by fluorescence melting measurements. The induced thermal stabilization on G4s, assumed by the target sequences in the same experimental conditions, is reported in Figure 1A and Supplementary Table S1. Data obtained were analyzed either in term of intensity of the thermal shift that must be high for *KIT*-related sequences to efficiently interfere with gene expression, and of selectivity G4 *vs* dsDNA, to reduce the risk of off-target effects.

Interestingly, some derivatives from the same scaffold showed a similar behavior. As an example, all the tested HAD derivatives recognized G4 irrespectively of DNA sequence (telomere, KIT1 or KIT2). However, NDI derivatives showed a preferential stabilization of the telomeric G4; therefore, they were not selected for further investigations. Within Phen derivatives, only their Ni(II) complexes, which contain two Phen moieties, were confirmed to be active as previously shown on the telomeric sequence; thus, we did not considered them suitable for *in cells* studies, since in the living environment the distribution among complexes with different stoichiometry can be hardly monitored.

On average, AQ derivatives showed higher thermal stabilization when compared to the tested AN derivatives; nevertheless, some variations in terms of efficiency and selectivity were highlighted in both families, according to the nature and relative position of side chains. This led us to consider them as promising candidates. To further reduce the number of potential hits, we added a second screening protocol, a G4 fluorescent intercalator displacement (G4-FID). This assay is based on the competitive displacement of thiazole orange (TO) from DNA by putative ligands.

In agreement with the existing literature we confirmed that, in our experimental conditions, TO showed a comparable binding constant for KIT1 and KIT2 [30]. Consequently, this assay provided a direct indication of the affinity ranking order of tested competitors for the target sequences. FID results are summarized in Figure 1B. Besides AQ and AN, we took into consideration a subset of HAD derivatives, selected for their good *KIT* thermal stabilization or selectivity. These compounds were reported to bind telomeric G4 according to different binding modes (end stacking or grooves insertion) [29]. No HAD derivative was able to displace the end-stacking agent TO from c-*KIT* promoter sequences. For AQ and AN derivatives, both FID and thermal stabilization data were in good agreement. On average, AQ derivatives were the best TO competitors; hence, we performed full titrations experiments with some derivatives. In particular, we focused on the comparison between 1,5 and 2,6 regioisomers within the AQ family (AQ1 and AQ5 vs AQ3 and AQ7, respectively), since they appeared to be the best performing compounds (Table 1). Within the 1,5 series, the aminoacidic composition of the side chain (i.e., βAla-Lys in AQ1 *vs* βAla-Phe-Lys in AQ5) did not cause significant variations in TO displacement; conversely, the insertion of a phenylalanine moiety within the 2,6 series (βAla-Lys in AQ3 *vs* βAla-Phe-Lys in AQ7) seemed to positively affect it. As far as AN derivatives are concerned, they were confirmed to be remarkably less efficient than AQs. Among them, AN6 was the best performing candidate on both *KIT* sequences. No differences were ever detected between KIT1 and KIT2 sequences.

**Table 1:** EC_50_^*a*^ (μM) derived from TO displacement (FID) by selected AQ and AN derivatives from G4 folded KIT sequences

	AQ1	AQ5	AQ3	AQ7	AN6
KIT1	0.32 ± 0.05	0.27 ± 0.02	0.50 ± 0.04	0.24 ± 0.04	4.11 ± 0.70
KIT2	0.35 ± 0.05	0.41 ± 0.03	0.62 ± 0.05	0.25 ± 0.02	3.63 ± 1.00

a ligand concentration that displaces 50% of TO from tested DNA sequences.

**Figure 2:**
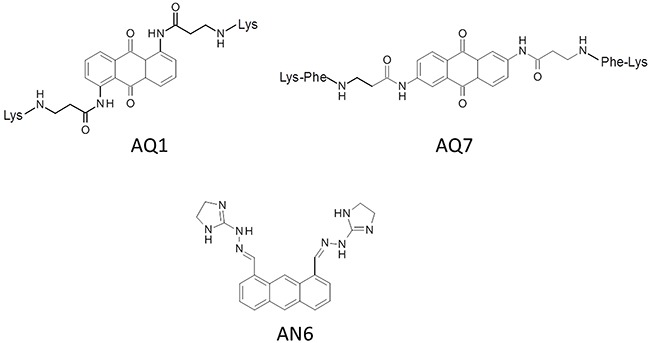
Chemical structures of selected compounds

Merging FID and thermal stabilization results, the anthracene derivative AN6 and anthracenedione derivatives AQ1 and AQ7 were therefore selected for further investigations (Figure 2).

### Binding affinity and functional interaction of selected ligands towards KIT1 and KIT2

**Figure 3:**
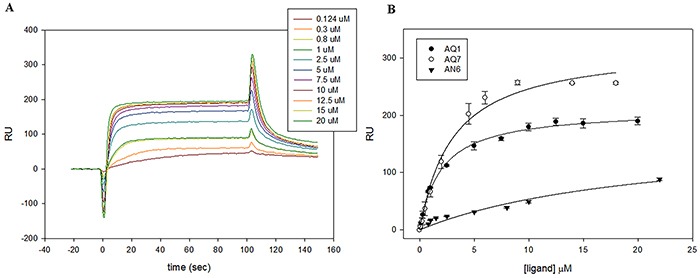
Representative examples of SPR analysis **A.** Sensorgrams derived from the analysis of AQ1 with KIT2. **B.** Plots of the RU at the steady state plotted vs the concentration of injected ligand on chip functionalized with KIT1.

To better characterize the interaction between the aforementioned ligands and the G4 folded form of KIT1 and KIT2, we performed Surface Plasmon Resonance (SPR) experiments. Oligonucleotides labeled at 5′ with Biotin-TEG (tetra-ethyleneglycol) were folded in KCl and subsequently immobilized on a gold chip functionalized with streptavidine. Sensorgrams were acquired and the data at steady state used to measure the binding constants of candidate binders towards *c-KIT* sequences (Figure 3, Table 2).

**Table 2:** Dissociation constants (Kd, μM) of selected AQ and AN derivatives with KIT sequences determined by SPR in 10 mM Tris, 50 mM KCl, pH 7.5, 0.025% surfactant P20

	KIT1	KIT2
AQ1	1.99 ± 0.15	1.01 ± 0.15
AQ7	3.04 ± 0.43	2.29 ± 0.28
AN6	71.5 ± 20.1	25.5 ± 4.01

All experimental equilibrium data were well fitted by a single binding model. Interestingly, all candidate ligands showed a preferential, albeit modest, interaction with KIT2. In line with results presented above, the AN6 binding constants were one order of magnitude lower than those provided by AQ derivatives.

**Figure 4:**
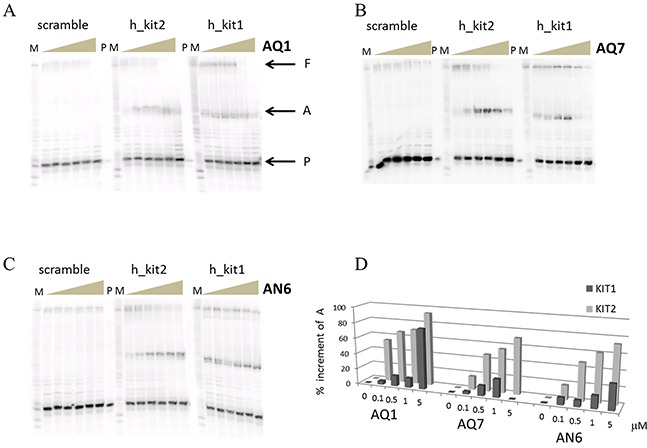
Polymerase stop assay performed with increasing concentration (0-10 μM) of AQ1 A. AQ7 B. or AN7 C Letters P, A and F respectively correspond to primer, arrest product and full length product, while M indicates the purine markers obtained according to the Maxam and Gilbert protocol from the full length product. In **D.** the quantification of the arrest product is reported. Errors were ± 10%.

Before moving toward the *in vitro* part of our study, we checked whether the binding of our ligands to *c-KIT* sequences actually impair the processing of *c-KIT* promoter; for this, we performed a polymerase stop assay. KIT1 and KIT2 sequences were inserted into a template strand and the elongation of a complementary primer by Taq polymerase was monitored. As shown in Figure 4, an increase of each ligand concentration in the reaction mixture resulted in a progressive reduction of the full length product; meanwhile, a predominant arrest product corresponding to the primer elongation up to the G-rich region appeared. In agreement with the above mentioned binding affinity ranking, this effect occurred at lower ligand concentration when the AQ derivatives were used, and a slight more pronounced efficiency toward *KIT2* sequence was noticed. This reinforces a model in which the G4-ligand complex can prevent *c-KIT* transcription.

### G4-ligands cytotoxicity

**Figure 5:**
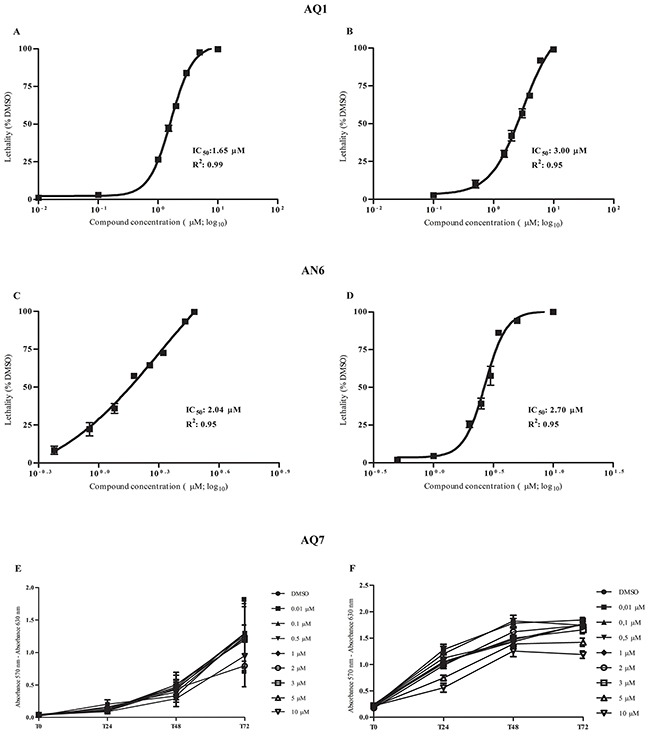
Dose-response and proliferation curves of HGC27 and MCF7 after treatment with the G4-ligands **A.** HGC27 and **B.** MCF7 dose-response curves from Alamar Blue experiments to determine IC_50_ values and R^2^ following the incubation with AQ1. **C.** HGC27 and **D.** MCF7 dose-response curves from Alamar Blue experiments to determine IC_50_ values and R^2^ after the exposure to AN6. **E.** HGC27 and **F.** MCF7 proliferation curves from sulforhodamine B experiments following the incubation with AQ7. Data are expressed as mean values ± standard deviation of three independent experiments, each one performed in different culture passages.

In short-term cultures (72 hours), AQ1 and AN6 showed dose-dependent cytotoxic effects in both MCF7 and HGC27 human cell lines. Dose-response curves, the relative IC_50_ values and the corresponding linear regression coefficients (R^2^), for each G4-ligand, are reported in Figure 5A-5D. By the Alamar Blue cytotoxicity test, AQ1 and AN6 dose-response curves identified lower IC_50_ values in HGC27 compared to MCF7 cell line. We could attribute such a difference to the different doubling time of the two cell lines (17 and 38 hours for HCG27 and MCF7, respectively). Indeed, IC_50_ values are usually lower in cell lines with a shorter doubling time, with a subsequent higher number of cell cycles in an equal period of time [31].

Derivative AQ7 was poorly cytotoxic, and the IC_50_ value could not be determined even using concentrations up to 10 μM (Figure 5E and 5F).

### Constitutive expression of target genes

**Figure 6:**
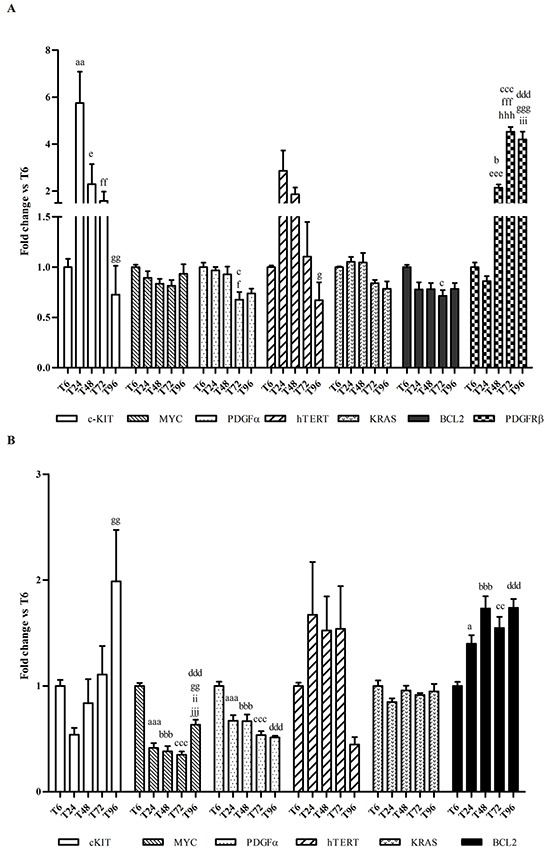
Effects of culturing time on gene expression **A.** Total RNA was isolated from HGC27 monolayers and mRNA levels of *c-KIT*, *MYC*, *PDGFA*, *hTERT*, *KRAS*, *BCL2* and *PDGFRβ* were measured by using a qPCR approach. **B.** Total RNA was isolated from MCF7 monolayers and mRNA levels of *c-KIT*, *MYC*, *PDGFA*, *hTERT*, *KRAS* and *BCL2* were measured by using a qPCR approach. Data (arithmetic means ± S.D.) are expressed as n-fold change (arbitrary units, a. u.) normalized to the RQ mean value of cells stopped at T6, to which an arbitrary value of 1 was assigned.^a, aa, aaa^: *P*<0.05; *P*<0.01; *P*<0.001 T_6_ vs T_24_; ^b, bbb^: *P*<0.05; *P*<0.001 T_6_ vs T_48_; ^c, cc, ccc^: *P*<0.05; *P*<0.01; *P*<0.001 T_6_ vs T_72_; ^ddd^: *P*<0.001 T_6_ vs T_96_; ^e, eee^: *P*<0.05; *P*<0.001 T_24_ vs T_48_; ^f, ff, fff^: *P*<0.05; *P*<0.01; *P*<0.001 T_24_ vs T_72_; ^g, gg, ggg^: *P*<0.05; *P*<0.01; *P*<0.001 T_24_ vs T_96_; ^hhh^: *P*<0.001 T_48_ vs T_72_; ^ii, iii^: *P*<0.01; *P*<0.001 T_48_ vs T_96_; ^jjj^: *P*<0.001 T_72_ vs T_96_.

To define the best protocol of exposure to selected G4-ligands, we measured the time-dependent changes (from T_6_ and up to T_96_) in the constitutive expression of *c-KIT* as well as of six other oncogenes containing G4 structures in their promoter region, i.e., *MYC, BCL2, PDGFA, PDGFRβ, KRAS* and *hTERT*. Results are shown in Figure 6. Overall, *c-KIT* showed a differential pattern of expression between the two cell lines. In HGC27, it reached a peak of expression at T_24_; then, it significantly decreased day by day. Conversely, in MCF7 cell line *c-KIT* expression increased slowly and reached a top at T_96_. As regards the other oncogenes, no time-dependent differences in constitutive gene expression were ever noticed with few exceptions, i.e. *BCL2* in MCF7 and *PDGFRβ* in HGC27, whose mRNA levels significantly increased with time. However, in MCF7 both *MYC* and *PDGFA* mRNAs decreased after T_6_, while the *PDGFRβ* gene was not detected.

Taking into consideration the aforementioned results, the aim of the present study and especially the role of *c-KIT* as the primary target, the transcriptional effects (stabilization of G4 structures and resulting decrease in gene transcription) of candidate G4-ligands were investigated in cells incubated for 6, 12 and 24 hours when *c-KIT* gene was mostly transcribed.

In the Supplementary Table S2 the experimental settings chosen for the whole set of qPCR and flow cytometry studies are reported.

### Evaluation of G4-ligands efficacy by qPCR and flow cytometry

**Figure 7:**
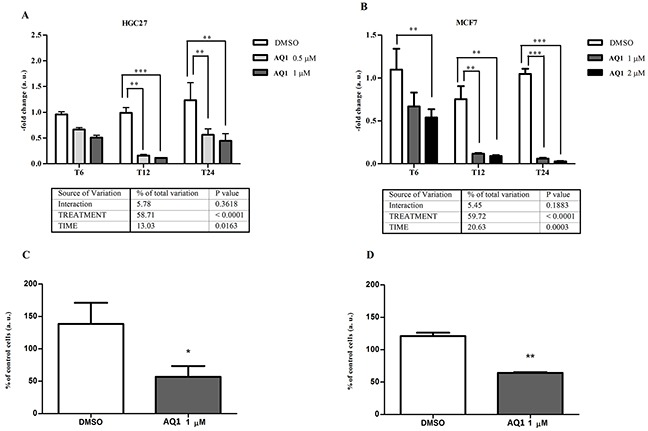
Effect of AQ1 on c-KIT mRNA and protein expression in HGC27 and MCF7 cell lines *c-KIT* mRNA levels **panels A-B.** were measured using a qPCR assay, and data (arithmetic means ± S.D.) are expressed as n-fold change (arbitrary units, a. u.) normalized to the RQ of control cells at each time of incubation (T_6_, T_12_, T_24_), to which an arbitrary value of 1 was assigned. The two-way ANOVA followed by Bonferroni post-test were used to check for statistical differences between doses and time of treatment. The c-kit protein amount **panels C-D.** was measured 48 hours post-exposure by flow cytometry, and data are expressed as n-fold change (%) of the mean fluorescence intensity (MFI) measured in untreated cells. The Student t-test was used to measure statistical differences between cells exposed to AQ1 and those treated with the vehicle (DMSO) only.^*,**, ***^: P<0.05; P<0.01; P<0.001.

In both cell lines, the incubation with AQ1 significantly (*P*<0.0001) downregulated *c-KIT* expression; namely, from two- up to ten-fold in HGC27 (Figure 7A) and thirty seven-fold in MCF7 cells (Figure 7B), in which the gene expression was almost completely suppressed after 24 hours of exposure.

Transcriptional results were confirmed, though to a lower magnitude, at the protein level by flow cytometry. A two-fold significant decrease (*P*<0.05) of c-kit amount was observed in HGC27, and a similar behavior was also noticed in MCF7 cell line (Figure 7C-7D, respectively). An example of flow cytometry dot plots, with population gate and histograms showing the fluorescence of CD117, is reported in Supplementary Figure S1.

**Figure 8:**
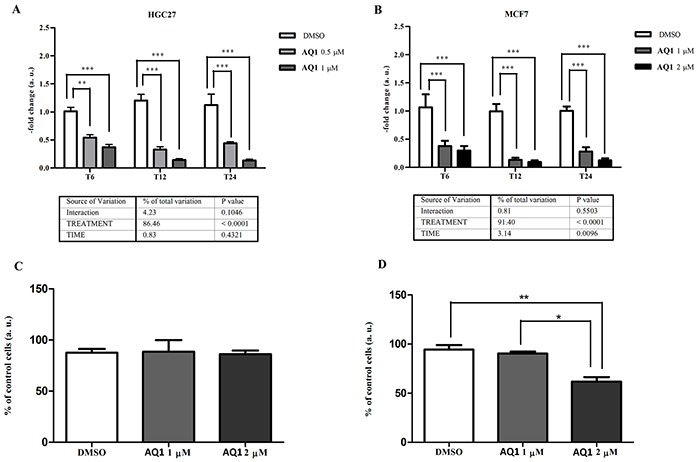
Effects of AQ1 exposure on *BCL2* mRNA and corresponding protein expression in HGC27 and MCF7 cell lines *BCL2* mRNA levels **panels A-B.** were measured using a qPCR assay, and data (arithmetic means ± S.D.) are expressed as n-fold change (arbitrary units, a. u.) normalized to the RQ of control cells at each time of incubation (T_6_, T_12_, T_24_), to which an arbitrary value of 1 was assigned. The two-way ANOVA followed by Bonferroni post-test were used to check for statistical differences between doses and time of treatment. The bcl-2 protein amount **panels C-D.** was measured 48 hours post-exposure by flow cytometry, and data are expressed as n-fold change (%) of mean fluorescence intensity (MFI), measured in untreated cells. The one-way ANOVA followed by Bonferroni post-test were used to identify statistical differences between cells exposed to AQ1 and those treated with the vehicle (DMSO).^*,**, ***^: P<0.05; P<0.01; P<0.001.

Besides *c-KIT*, AQ1 caused also a significant (*P*<0.0001) inhibition of *BCL2* gene expression in both cell lines (Figure 8A-8B), whose magnitude was three to five-fold and four to six-fold for HGC27 and MCF7, respectively. No post-transcriptional effects were noticed in HGC27 (Figure 8C); conversely, a significant (*P*<0.01) decrease of bcl-2 protein was observed, at 48 hours, in MCF7 cells exposed to 2 μM AQ1 (Figure 8D).

In other investigated oncogenes with G4 structures in their promoter region a significant, although of minor importance, downregulation was observed for *PDGFRβ* in HGC27 (*P*=0.0003: Supplementary Figure S2) and for *MYC* and *hTERT* in MCF7 (*P*<0.0001: Supplementary Figure S3).

Regarding AN6, it never affected *c-KIT* mRNA in HGC27 cell line and an overall absence of modulation was also observed in MCF7 cell line, except for a decrease, only at T_12_ hours and at the highest concentration (*P*<0.05; Supplementary Figure S4A-S4B). After 48 hours of exposure, the c-kit protein amount was significantly decreased (*P*<0.05 and *P*<0.01 in HGC27 and MCF7, respectively; Supplementary Figure S4C-S4D).

Contradictory results were obtained for the other oncogenes. *PDGFA* mRNA levels increased after AN6 treatment in both cell lines (*P*<0.0001; Supplementary Figure S5D and S6D), while *KRAS* and *PDGFRβ* were up-regulated only in HGC27 cells (*P*<0.0001; Supplementary Figure S5C and S5E). On the other hand, a significant downregulation of *MYC* and *hTERT* mRNA were noticed in MCF7 cells exposed to the highest AN6 concentration (*P*<0.0001; Supplementary Figure S6A-S6B, respectively).

Finally, the exposure to AQ7 at 10 μM did not lead to a significant up- or downregulation of *c-KIT* mRNA as well as of the whole set of other investigated oncogenes (data not shown). This well relates to its poor cytotoxic profile but not to its affinity for the promotorial c-KIT G4, thus suggesting a low ability of AQ7 in reaching this intracellular target.

### Confirmatory results with other cellular models

**Figure 9:**
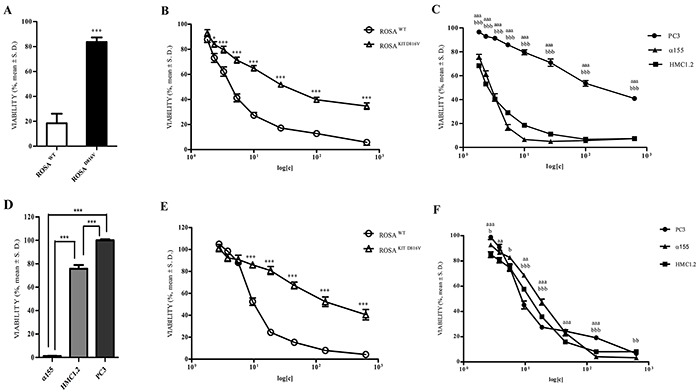
Effect of exposure (72 hours) to Imatinib, AQ1, and AN6 on cell proliferation of ROSA^WT^, ROSA^KITD816V^, α155, HMC1.2 and PC3 cell lines Data are expressed as percentage of viability ± S.D. against cells treated only with the vehicle (DMSO). **A.** Effect of Imatinib 1 μM on ROSA cell lines. Student *t*-test. ^***^: *P*<0.001. **B.** Effect of AQ1 on ROSA cell lines. Student *t*-test. ^*, ***^: *P*<0.05; *P*<0.001. **C.** Effect of AQ1 on α155, HMC1.2 and PC3. Student *t*-test. ^aaa^: *P*<0.001 α155 vs PC3; ^bbb^: *P*<0.001 HMC1.2 vs PC3. **D.** Effect of Imatinib 1 μM on α155, HMC1.2 and PC3 cell lines. One-way ANOVA followed by Bonferroni post-test. ^***^: *P*<0.001. **E.** Effect of AN6 on ROSA cell lines. Student *t*-test. ^***^: *P*<0.001. **F.** Effect of AN6 on α155, HMC1.2 and PC3. Student *t*-test. ^aa^, ^aaa^: *P*<0.01; *P*<0.001 α155 vs PC3; ^b, bb, bbb^: *P*<0.05; *P*<0.01; *P*<0.001 HMC1.2 vs PC3.

To confirm that the effective molecular target of AQ1 were the G4 sequences in *c-KIT* promoter, a proliferation study was undertaken in the SCF-dependent ROSA cell line (a human mast cell line); in particular, the wild-type cell line (ROSA^WT^) and its SCF-independent sub-clone ROSA^KITD816V^, engineered by lentiviral transfection [32] and regulated by a different promoter. In Figure 9A, the results obtained treating cells with 1 μM imatinib as a control of stable transfection are reported. As expected, ROSA^WT^ cells were much more sensitive to the TKI than the sub-clone transfected with the mutation KITD816V, which confers resistance to imatinib.

When we compared the AQ1 dose-response curves in the aforementioned cell lines, a significant inhibition of cell proliferation was observed only in ROSA^WT^ cells (Figure 9B).

The antiproliferative effects of AQ1 were then tested in other three cell lines; α155 and HMC1.2 cell lines, in which the cell growth strictly depends on *c-KIT*, and PC3 cell line where *c-KIT* is not constitutively expressed [33]. When compared with PC3 cell line, an overall and significant inhibition of cell proliferation was observed in α155 and HMC1.2 cell lines (Figure 9C). The same experiment was also repeated on other cell lines where *c-KIT* is not responsible for growth, i.e. TOV112 ovarian cancer cells and KARPAS299 lymphoma cell line and data corroborated those obtained with PC3 cell line (data not shown).

Whether no differences were noticed between α155 and HMC1.2 cell lines following AQ1 exposure, a differential response was visible after treatment with imatinib between imatinib-sensitive (α155) and imatinib-resistant (HMC1.2 and PC3) cell lines (Figure 9D). Overall, these results suggest that AQ1 effectively binds to *c-KIT* promoter, albeit the transcriptional downregulation observed for *BCL2*, prevent us to define such a binding as specific.

Likewise to AQ1, AN6 seemed to bind the *c-KIT* promoter (Figure 9E). Indeed, a different pattern of inhibition between ROSA^WT^ and ROSA^KITD816V^ cells proliferation was noticed. Nevertheless, limited differences were noticed between PC3, α155 and HMC1.2 cell lines dose-response curves (Figure 9F). This might suggest a non-selective action of AN6 towards other G4 sequences, particularly when *c-KIT* gene is barely expressed or undetectable. Taking into account these results as a whole (including the reduced inhibitory effect on *c-KIT* mRNA), we focused our attention on AQ1.

**Figure 10:**
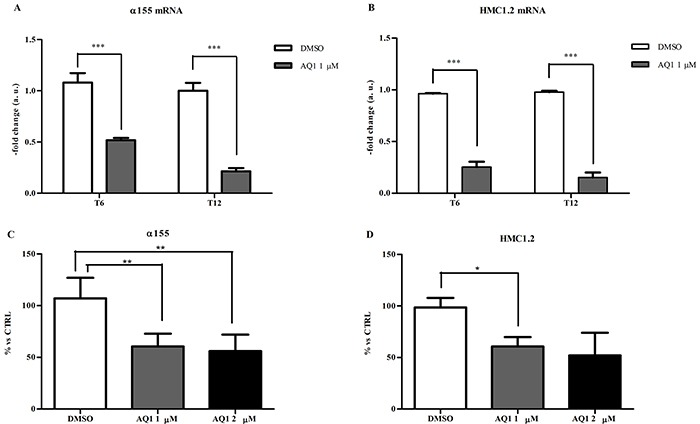
Effect of AQ1 on *c-KIT* mRNA and protein expression in α155 and HMC1.2 cell lines The *c-KIT* mRNA levels **panels A-B.** were measured using a qPCR assay, and data (arithmetic means ± S.D.) are expressed as n-fold change (arbitrary units, a. u.) normalized to the RQ of control cells at each time (T_6_, T_12_) to which an arbitrary value of 1 was assigned. Two-way ANOVA followed by Bonferroni post-test was used to assess statistical differences between doses and time of treatment. ^***^: significant differences (*P*<0.001). The c-kit protein amounts **panels C-D.** were measured 48 hours post-exposure by flow cytometry and data are expressed as n-fold change (%) to the mFI of not treated cells. One-way ANOVA followed by Bonferroni post-test was used to assess statistical differences between cell treated with AQ1 and those treated with the vehicle (DMSO). ^*,**^: P<0.05; P<0.01.

Following the treatment of α155 and HMC1.2 cell lines with 1 μM AQ1 (final concentration), we measured *c-KIT* mRNA and protein by qPCR and flow cytometry, respectively. A significant transcriptional downregulation was noticed, in both cell lines, after 6 and 12 hours of exposure (Figure 10A-10B). This result was confirmed at the post-translational level after 48 hours of exposure (Figure 10C-10D). An example of scatter plots and histograms obtained with α155 cell line is reported in Supplementary Figure S7. Following the treatment with AQ1, the side-scatter of cell population changed, and such a phenomenon could be attributed to a morphological effect. To demonstrate that the inhibition of c-kit protein was not a toxic effect, we performed the same experiment labeling the HLA complex, a protein supposed not being influenced by the treatment. Data obtained with α155, HMC1.2 and KARPAS299 cell lines (this latter survives independently from *c-KIT*) showed that HLA complex expression was never influenced by AQ1 exposure (Supplementary Figure S8). An example of scatter plots and histograms obtained with α155 cell line is reported in Supplementary Figure S9. Overall, this would confirm that occurring morphological changes were not due to a non-selective toxicity of AQ1.

## DISCUSSION

*c-KIT* silencing is currently a promising therapeutic approach in oncology. To this purpose, the presence of putative G4 structures within *c-KIT* promoter represents an attractive target for G4-ligand design. If selective, these compounds are expected to alter *c-KIT* regulation at the transcriptional level, with pharmacologically useful post-translational effects. The advantages of such an approach rest in the structural knowledge of the target (NMR and X-ray data of the G-rich promoter region of *c-KIT* are currently available) [34-38] and on the small size of the potential ligands for these targets (thus allowing affordable subsequent optimization steps). To clarify the molecular features required by a G4-ligand (a) to bind the selected KIT1 and KIT2 sequences and, (b) to consequently cause the downregulation of *c-KIT* expression in cancer cells we started with the screening of a library of compounds.

As regards the target recognition step, the combination of two different binding assays (thermal stabilization of the G4 form of KIT1/KIT2 and the displacement of a G4-bound dye) allowed us to identify three potential candidates out of thirty-nine tested. They belonged to the AQ or AN series. Interestingly, the binding affinity was comparable to the better performing *KIT*-targeted agents, thus supporting the tricyclic aromatic structure as a suitable scaffold to recognize the selected nucleic acid sequences. Nevertheless, before labeling them as promising hits, an evaluation of their behavior at the cellular level was clearly required.

In most of publications focused on *c-KIT* and G4s, the MCF7 and HGC27 cell lines were used as experimental *in vitro* models [12, 20, 22, 39] thus justifying the choice to test our candidate G4-ligands on these cells.

Although the affinity of the two AQ derivatives for the target sequences was comparable, AQ7 showed neither antiproliferative effects nor *c-KIT* downregulation up to a concentration of 10 μM. Consequently, we decided to exclude it from following investigations.

Overall, the most interesting results were obtained with AQ1, which caused a significant inhibition of *c-KIT* mRNA levels in both cell lines; furthermore, such a transcriptional downregulation was confirmed at the protein level by flow cytometry. Worth mentioning, the present mRNA downregulation is one of the highest ever recorded in studies about the transcriptional effects of candidate ligands targeting the G4 structures of *c-KIT* promoter [12, 20, 22, 39]. This transcriptional effect was more pronounced in MCF7 than in HGC27 cells, and this could be due to the different pattern of constitutive expression of *c-KIT* in the two cell lines. An analogous behavior has already been observed by Bejugam *et al*. in a similar study and with a different G4-ligand [20].

The mechanism of action of AQ1 was further validated in *in vitro* models commonly used to study the effects of TKIs on *c-KIT* expression and its mutational status [32, 40]. At first, the experiment with ROSA cells indirectly demonstrated that the observed inhibition of cell proliferation was effectively due to the AQ1 binding to *c-KIT* promoter. Furthermore, the α155 and HMC1.2 cell lines, whose growth strictly depends on *c-KIT*, showed a high sensitivity to AQ1 while the prostate cancer cell line PC3 did not. All this further results confirm that AQ1 binds to *c-KIT* promoter. The PC3 cell line lacks the proto-oncogene [33], and the minor inhibition of cell proliferation supports the absence of a strong functional binding to other G4 structures. This is important to potentially rule out severe off-target effects. Nevertheless, it must be taken into account that several G4 ligands selective for one sequence in cell-free models, inhibited cell growth by modulating at the same time the expression of other genes [41, 42]. For this reason, we decided to check for possible ligand interactions with other oncogenes whose promoter contained one or more putative G4 sequences. In particular, AQ1 was shown to downregulate *BCL2* expression in both MCF7 and HGC27, and such a finding was confirmed also in α155 and HMC1.2 cell lines (data not shown). It was already reported that some anthraquinones from natural sources decrease *BCL2* expression and promote apoptosis [43], and our results confirm such an evidence, despite the less important inhibition noticed at the protein level. This latter event has already been reported for other G4-ligands [44]. However, it has been documented that SCF, the endogenous *c-KIT* ligand, maintains the survival of human mast cells by repressing apoptosis through the expression of *BCL2* [45]. Therefore, it is actually impossible to attribute the observed transcriptional effect to a direct interaction of AQ1 with the *BCL2* G-rich regions or to an indirect inhibition resulting from the decrease of *c-KIT* mRNA. Further molecular investigations are currently underway to better clarify this open question.

Worth mentioning, in the present study we also demonstrated that HMC1.2, a cell line naturally possessing the *c-KIT* mutation D816V and thus resulting resistant to imatinib, was highly responsive to AQ1. It is well known that some *c-KIT* mutations could represent a limitation in the use of TKIs, i.e. mutations of *c-KIT* exon 17 such as D816V and occurring in ~80% of adult mastocytosis, and some mutations of *c-KIT* exon 9 in GIST [9, 46]. Present results are therefore encouraging and unveil the potentialities of this compound to be effective also against tumors with any type of *c-KIT* mutation in the coding region. The decrease of *c-KIT* mRNA and protein, noticed in MCF7 and HGC27, were also confirmed on α155 and HMC1.2 cell lines: to the best of our knowledge this is the first screening of candidate G4-ligands undertaken in a panel of designed target-specific cell lines and ever published so far.

In line with *in solution* data, the light antiproliferative effect observed in ROSA^KITD816V^ cells confirmed that AN6 binds to *c-KIT* promoter. However, the proliferation studies executed on our panel of cell lines showed a common and similar inhibition of cell proliferation, independently from their high or low *c-KIT* expression.

At the same time, exposure of MCF7 and HGC27 cell lines to AN6 caused a minor effect on *c-KIT* mRNA whereas c-kit protein was markedly inhibited. This result could be justified either with the lower affinity of AN6 toward the G4 structures within *c-KIT* promoter, or with concomitant effect of AN6 on distinct molecular pathways. Indeed, in the present study a downregulation of *MYC* and *hTERT* mRNAs and an increase in *KRAS, PDGFA* and *PDGFRβ* gene expression were noticed. A possible up-regulation of other genes beyond the target one, following the exposure to candidate G4-ligands, has already been described [47]. Overall, more detailed studies are needed to further decipher the particular molecular mechanisms occurring following G4-mediated changes in oncogene transcription. However, since *c-KIT* was the main target of the present study, AN6 was not considered as a good candidate.

In conclusion, this work exhaustively demonstrated the capability of a G4-ligand, i.e. AQ1, to downregulate *c-KIT* mRNA and c-kit protein amounts and, consequently, to block proliferation in different but target-designed *in vitro* models. Present results constitute a solid starting point for further studies about this promising molecule; among them, studies aiming to demonstrate the specific binding of AQ1 to G4 sequences of *c-KIT* promoter and to elucidate the exact molecular mechanisms of AQ1-dependent cell damage.

## MATERIALS AND METHODS

### Ligands

AQ and AN derivatives were synthesized by Prof. G. Zagotto (University of Padua, Italy); NDIs were synthesized by Prof. V. Tumiatti and A. Milelli (University of Bologna, Italy), HADs by Prof. D. W. Boykin (Georgia State University, USA) and Phen analogues by Prof. A. P. Krapcko (University of Vermount, USA). Schematic drawings of all the tested compounds are shown in the supplementary materials (Supplementary Schemes S1-S5). Stock solutions (1 mM) of each library member were prepared in dimethyl sulfoxide (DMSO, Sigma-Aldrich Co., St. Louis, USA) and stored at −20°C. Stock solutions (10 mM) of selected ligands AQ1, AQ7 and AN6 were prepared in DMSO and freshly diluted in culture medium the day of the experiment.

### Fluorescence melting assay

Fluorescence melting analyses were performed with a Roche Light Cycler® 480 II (Roche Applied Science, Indianapolis, USA), using an excitation source at 488 nm and recording the fluorescence emission at 520 nm. Samples (20 μl final volume) containing 0.25 μM DNA were loaded on a 96-well plate in 10 mM LiOH pH 7.5 with H_3_PO_4_, containing 50 mM KCl and increasing concentrations of ligands. Samples were first heated to 95°C at a rate of 0.1°C/s, maintained at 95°C for 5 min and then annealed by cooling to 30°C at a rate of 0.1°C/s. Then samples were maintained at 30°C for 5 min before being slowly heated to 95°C (1°C/min) and annealed at a rate of 1°C/min. During these slow steps no hysteresis was observed. For the analyses with double strand oligonucleotide, the two complementary strands were annealed before ligand addition and melting acquisition. Each curve was repeated at least three times and errors were ± 0.4°C. Melting temperatures were determined from the first derivatives of the melting profiles using the Roche Light Cycler software (Roche Diagnostics, Mannheim, Germany).

### Fluorescent intercalator displacement assay

FID screening assay was performed in a 96-well plate reader Victor3TM 1420 Multilabel Counter Perkin Elmer (Perkin Elmer, Waltham, USA) set at 25°C. An excitation λ of 485 nm and emission λ of 535 nm were used. Before data acquisition the 96-well plate was shaken for 2 s. In each well 80 μl of reaction mixture containing oligonucleotide (1 μM), Thiazole Orange (TO, Sigma-Aldrich Co., St. Louis, USA) (2 μM) and increasing concentrations of each compound (1-2-8 μM) in 10 mM Tris, 50 mM KCl, pH 7.5 were loaded. Fluorescence titrations were performed in a Perkin Elmer LS55 Luminescence spectrometer (Perkin Elmer, Waltham, USA) equipped with a cell holder termostated at 25°C and using an excitation wavelength of 501 nm. For FID, a solution containing 0.62 μM of target DNA and 1.24 μM of TO was added of increasing concentrations of tested compounds in 10 mM Tris, 50 mM KCl, pH 7.5. The percentage of TO displacement was calculated as TO displacement = 100 - [(F/F_0_) × 100], where F_0_ is the fluorescence in the absence of ligand and F the fluorescence recorded at each point of titration. TO displacement was plotted as a function of compound concentration. From these plots the EC_50_ (half maximal effective concentration) was calculated. Each titration was repeated at least in triplicate.

### Surface plasmon resonance

SPR measurements were performed on a Biacore X100 (GE Healthcare Life Sciences, Little Chalfont, United Kingdom) set up with a streptavidine-coated sensor chip prepared for use by conditioning with injections of 1 M NaCl, 50 mM NaOH in 50% isopropanol for 1 min and finally extensively washed with a 0.22 μm filtered buffer (10 mM Tris pH7.5, 50 mM KCl, 0.025% P20). Previously annealed, 5′-biotinylated oligonucleotides were then immobilized on one cell of the chip surface by flowing a 50 nM DNA solution at a 1 μL/min flow rate until the level of 400 response unit (RU) was obtained. A second cell was left blank as control. Sensorgrams were acquired using serial dilution of tested ligands in the same buffer. To avoid interference by DMSO, its concentration was kept constant and added to the running buffer too (1.7%). Compounds solutions were injected at a 25 μL/min flow rate until a constant steady-state was reached (60-200 s). After each run, a 30 s regeneration step was performed with 10 mM glycine pH 2.5 followed by a 60 s stabilization period in the running buffer. The experimental RU values were recorded at the steady state. Data were fitted according to a binding site model.

### Polymerase stop assay

A 20 nM equimolar mixture of ^32^P 5′-labeled primer and the human telomeric template sequence HT4-temp d[TC_2_A_2_CTATGTATAC(T_2_AG_3_)_4_ACATATCGATGA_3_T_2_GCTATAGTGAGTCGTAT_2_A] was annealed in the required polymerase buffer and subsequently added of increasing candidate ligand concentrations. After incubation (30 min at room temperature), 2.5 U of Taq polymerase (Thermo Scientific, Waltham, USA) and 100 μM dNTPs mixture were added to each sample and the resulting solutions were kept for 30 min at 55°C. Reaction products were resolved by gel electrophoresis (12% polyacrylamide gel with 7 M urea) in 1X TBE (89 mM Tris base, 89 mM boric acid, 2 mM Na_2_EDTA). Gels were dried and resolved bands were visualized on a PhosphorImager (GE Healthcare, Little Chalfont, United Kingdom).

### Cell cultures

The breast adenocarcinoma human cell line MCF7 (Leibniz Institute DSMZ-German Collection of Microorganisms and Cell Cultures) and the human gastric carcinoma cell line HGC27 (European Collection of Cell Cultures) were maintained in 25 or 75 cm^2^ flasks under a humidified 5% CO_2_ atmosphere, at 37°C. Cells were grown in Eagle's Minimal Essential Medium (EMEM, Gibco^®^ Life Technologies, Carlsbad, USA) supplemented with 10% fetal bovine serum (Gibco^®^ Life Technologies, Carlsbad, USA), 2 mM L-glutamine (Euroclone, Milan, Italy), 1% non-essential amino acids (Euroclone, Milan, Italy) and 1% penicillin/streptomycin (Euroclone, Milan, Italy). Human insulin (10 μg/mL) (Elli Lilly & Co., Indianapolis, USA) was also added to MCF7 cell culture medium.

The human mast cell leukemia HMC1.2, containing both juxtamembrane and catalytic *c-KIT* domain mutations (V560G and D816V), was kindly provided by Dr. Joseph Butterfield (Mayo Clinic, Rochester, MN, USA). This cell line, as well as the human mast cell leukemia α155 (possessing only the V560G mutation), the human prostate cancer cell line PC3, the human lymphoma cell line KARPAS 299 and ROSA mast cell lines (wild type and transfected with KITD816V) [32] were cultured in RPMI medium (Gibco^®^ Life Technologies, Carlsbad, USA) supplemented with 10% FBS, 2 mM L-glutamine and 1% penicillin/streptomycin.

Cell number and viability were checked by using Trypan Blue dye exclusion test (Sigma-Aldrich Co., St. Louis, USA). For all the experiments, cells were used from passage 5 to passage 25 maximum. Furthermore, cell cultures were checked for Mycoplasma contamination both before and at the end of experiments through PCR Mycoplasma Test Kit (PromoKine, Heidelberg, Germany).

### G4-ligands cytotoxicity

MCF7 and HCG27 cells were seeded at concentrations comprised between 0.3×10^4^ and 0.5×10^4^ cells/well in a 96-well flat bottom plate (Sarstedt Italia, Verona, Italy). After 24 hours, AQ1 or AN6 were added at concentrations from 0.01 μM up to 10 μM for 72 hours. Additional wells exposed either to the vehicle (DMSO, 0.1% final concentration) or to medium alone were prepared, too. At the end of the experiment, 20 μL of CellTiter-Blue^®^ Cell Viability Assay (Alamar Blue, Promega, Madison, USA) were added to each well and the fluorescence was measured at 560 nm as excitation wavelength and 590 nm as emission wavelength, by using a VICTOR^™^X4 Multilabel Plate Reader (Perkin Elmer, Waltham, USA). Three separate experiments were executed and each concentration was tested in sestuplicate. In line with preliminary comparative *in vitro* studies (data not shown), the sulforhodamine B (Sigma-Aldrich Co., St. Louis, USA) assay was used to measure the effect of AQ7 on cell proliferation. Both cell lines were exposed to a range of concentrations up to 10 μM for 0, 24, 48, 72, and 96 hours. Three separate experiments were executed, and each concentration was tested in sestuplicate.

### Target genes constitutive expression

No information about *c-KIT* constitutive expression and its variation as a function of time were available in literature; therefore, a first set of experiments were undertaken to define the best experimental settings for measuring G4-ligands efficacy.

Cells were seeded onto 6-well plates at concentration of 5×10^5^ and 4×10^5^ cells/well (for MCF7 and HGC27, respectively) and collected after 6, 24, 48, 72 and 96 hours. Monolayers were washed with 1 mL of fresh PBS, scraped off and centrifuged at 100*g* for 5 min. Cell pellets were resuspended in 0.5 mL of TRIzol^®^reagent (Invitrogen^™^, Life Technologies, Carlsbad, USA), and total RNA was extracted according to manufacturer's instructions. Nucleic acids yield and purity (260/280 and 260/230 nm absorbance ratios) were measured by using the Nanodrop ND-1000 Spectrophotometer (Nanodrop Technologies, Wilmington, UK), whilst their quality was checked by 1% agarose gel electrophoresis. Total RNA (1 μg) was reverse transcribed by using the High Capacity cDNA Reverse Transcription Kit (Life Technologies, Foster City, USA) and following the manufacturer's instructions.

The full list of primers used in the present study for qPCR analysis is reported in Supplementary Table S3. Apart from *c-KIT*, we analyzed other 6 oncogenes known to contain putative G4 structures in their promoter region: the V-Myc Avian Myelocytomatosis Viral Oncogene Homolog (*MYC*), the Kirsten rat sarcoma viral oncogene homolog (*KRAS*), the beta-type platelet-derived growth factor receptor (*PDGFRβ*), the B-cell lymphoma 2 (*BCL2*), the Platelet-Derived Growth Factor Alpha Polypeptide (*PDGFA*) and the Telomerase Reverse Transcriptase (*hTERT*). Primers for *MYC, PDGFA, BCL2* and *PDGFRΔ* were obtained from previously published studies [22, 48-50], and the most appropriate Universal Probe Library (UPL) probe was identified using the UPL Assay Design Centre web service (Roche Diagnostics, Mannheim, Germany). For the other oncogenes, primers were designed *ex novo* using the Primer3 software (http://primer3.ut.ee/). Assay specificity was evaluated *in silico* using the BLAST tool as well as experimentally with Power SYBR Green I (Life Technologies, Carlsbad, CA) amplification and melting curve analysis.

Quantitative real-time RT-PCR (qPCR) reactions (10 μL final volume) consisted of 1X LightCycler 480 Probe Master (Roche Applied Science, Indianapolis, USA), 300 or 600 nM forward and reverse primers (Eurofins MWG Operon, Ebersberg, Germany) derived from the assay set-up, 200 nM human UPL probe (Roche Applied Science, Indianapolis, USA) and 2.5 μL of 1:7.5 diluted cDNA. The analysis was performed in a LightCycler 480 Instrument (Roche Applied Science, Indianapolis, IN) using standard PCR conditions (95°C for 10 min; 45 cycles at 95°C for 10 s and at 60°C for 30 s; 40°C for 30 s). Calibration curves, using 3-fold and 4-fold serial dilutions of a cDNA pool, were performed, and corresponding values of slope, efficiency (E) and dynamic range, for each cell line, are reported in Supplementary Table S4. Only qPCR assays with E (%) comprised between 90% and 110% were considered acceptable. qPCR data were analyzed with the LightCycler480 software release 1.5.0 (Roche Applied Science, Indianapolis, USA) and using the second derivative method. The mRNA relative quantification (RQ) was performed by using the ΔΔCt method [51]. Three internal control genes (ICGs), e.g. Hypoxanthine Phosphoribosyltransferase 1 (*HPRT1*), Glyceraldehyde-3-Phosphate Dehydrogenase (*GAPDH*) and Beta-2-Microglobulin (*Δ2M*) were amplified in all samples, but only ICGs genes whose expression did not vary during the experimental conditions were considered for RQ. A cDNA pool was used as calibrator. Experiments were performed in triplicate and, for each experiment, two biological replicates were included.

### Determination of G4-ligands efficacy by qPCR

To measure the transcriptional effects of each candidate G4-ligand, cells (24 hours after seeding) were incubated either with vehicle (DMSO, 0.1% final concentration) or two sub-cytotoxic ligand concentrations (1/3 and 2/3 of the corresponding IC_50_ value). According to data about the effect of time on *c-KIT* constitutive expression, cells were collected as described above after 6, 12 and 24 hours of incubation. Methods used for RNA extraction, reverse transcription and qPCR were the same described in the previous paragraph. ICGs expression was checked within every experimental condition. The choice of the most suitable ICGs to be used for normalization was cell line- and ligand-dependent.

### Determination of G4-ligands efficacy by flow cytometry

Cells (5×10^5^/well and 4×10^5^/well for MCF-7 and HGC-27 cell lines, respectively) were seeded in 6-well plates; after 24 hours, the vehicle (DMSO, 0.1% final concentration) or AQ1/AN6 (1 μM, final concentration) were added to the medium. Forty-eight hours post-exposure, monolayers were washed twice with PBS 1X 0,02% EDTA, detached and centrifuged at 100*g* for 4 min. Cells were resuspended in RPMI medium (Gibco^®^ Life Technologies, Carlsbad, USA) supplemented with 3,3% FBS (Gibco^®^ Life Technologies, Carlsbad, USA). Fifty μL of the cell suspension were incubated for 15 min at 4°C with 50 μL of a rat anti-mouse monoclonal antibody raised against cell surface c-kit (CD117_PE,_ clone ACK 45 BD Pharmingen, California, USA) concentrated 1:25; then, a wash step with 500 μL of PBS followed by centrifugation at 100*g* at 4°C for 10 min were performed. After removing the supernatant, 900 μL of PBS 1X were added to the cells. For bcl-2 detection, 100 μL of the cell suspension were fixed and permeabilized with the IntraStain kit (DAKO Italia SRL, Milano, Italy) and then incubated with an anti-BCL2 antibody FITC-conjugated (clone 124, DAKO Italia SRL, Milano, Italy). For acquisitions, the CyFlow® Space (Partec® GmbH, Münster, Germany) was used. Cells not incubated with the anti-CD117_PE_ and anti-BCL2_FITC_ were considered as negative controls. For each sample, c-kit expression was evaluated both in terms of events that stained for CD117 and in terms of mean fluorescent intensity (MFI), calculated as the ratio of the MFI in neoplastic cells by the MFI of unstained cells. Samples were analyzed by using FlowMax® software (Quantum Analysis GmbH, Münster, Germany), version 2.82.

### Confirmatory experiments with other cellular models

Confirmatory proliferation studies were executed on α155, HMC1.2, PC3, ROSA^WT^ and ROSA^KITD816V^ cell lines, using methods and conditions mentioned above (IC_50_ determination). Cells were treated with AQ1 or AN6 at concentrations from 0.2 μM up to 3 μM and for 72 hours. To check for the resistance or sensitivity of the used cellular models, an imatinib mesylate control (1 μM final concentration) was included in the experimental setting.

As regards qPCR, three independent confirmatory experiments were executed in α155 and HMC1.2 cell lines to confirm the transcriptional effects of AQ1 on *c-KIT* mRNA. About 9×10^5^/well cells were seeded onto 6-well plates, and DMSO or AQ1 were added at final concentrations of 0.1% and 1 μM, respectively. Cells were harvested 6 and 12 hours of incubation and centrifuged at 100*g* for 5 min; pellets were then washed once with 1 mL PBS and, finally, submitted to the same methodological procedure reported above (determination of G4-ligands efficacy by qPCR). For each cell line, values of slope, efficiency and dynamic range of qPCR assays are reported in Supplementary Table S5.

For confirmatory flow cytometry investigations, HMC1.2, α155 and KARPAS 299 cells were seeded in P6-well plates (3×10^5^ cells/well); then, DMSO or AQ1 were added at final concentrations of 0.1%, and 1 or 2 μM, respectively. After 48 hours, 3×10^5^ cells were collected. HMC1.2 and α155 cells were labeled, at 4°C for 30 min, with mouse monoclonal anti CD117 SC 13508 (Santa Cruz Biotech, Texas, USA), diluted 1:100. The secondary antibody used was an anti-mouse PE conjugated (diluted 1:50). The high affinity IgE receptor (FcεRI), present on mast cell membrane, was saturated by incubation with human serum for 10 min at room temperature. The human leukocyte antigens (HLA) were used as reference proteins and α155, HMC1.2 and KARPAS 299 cell lines were labeled with monoclonal anti-human leukocyte antigen (HLA) PeCy5 conjugated (W6-32 eBioscience, California, USA). Unstained cells with the proper isotype control were used to check for non-specific fluorescence signals.

The cytofluorimetric analysis was made in a BD LSRFortessa™ (Becton Dickinson, New Jersey, USA) and data were analyzed by using DIVA^TM^ (BD Pharmingen, California, USA) software. The c-kit expression was evaluated, for each sample, in terms of MFI, calculated as the ratio of MFI in neoplastic cells by the MFI of unstained cells. Final results consisted in the mean of three different experiments.

### Statistical analysis

Data statistical analysis was performed using GraphPad Prism version 5.00 for Windows (GraphPad Software, San Diego, USA). The IC_50_ values were determined by nonlinear regression analysis, fitting a sigmoid dose-response curve.

Data on the time-dependent variation in target gene constitutive expression were expressed as -fold change of the respective T_6_ value, and analyzed using one-way analysis of variance (ANOVA) followed by the Bonferroni's post-test.

The presence of statistically significant differences in mRNA levels in cells treated with candidate G4-ligands were checked using the two-way ANOVA followed by Bonferroni's post-test; this approach allowed us to verify if any difference in term of transcriptional response was either dose and/or time-dependent. Each RQ value of treated cells was normalized to the average RQ of the respective time-control samples.

In cell proliferation experiments, the obtained viability data were analyzed with the Student t-test.

Data from cytofluorimetric analysis were expressed as n–fold changes against control cells; either the Student *t*-test or the one-way ANOVA were used to unveil statistically significant differences between cells treated with vehicle only and AQ1.

## SUPPLEMENTARY SCHEMES, FIGURES AND TABLES


