# Supplementary material for: Screening of candidate G-quadruplex ligands for the human c-KIT promotorial region and their effects in multiple in-vitro models
Source: Oncotarget. 2016 Mar 1;7(16):21658–75. doi: 10.18632/oncotarget.7808 (PMC5008313; doi:10.18632/oncotarget.7808)
Supplement: Supplementary file 1 [file oncotarget-07-21658-s001.pdf]

SUPPLEMENTARY SCHEMES, FIGURES AND TABLES

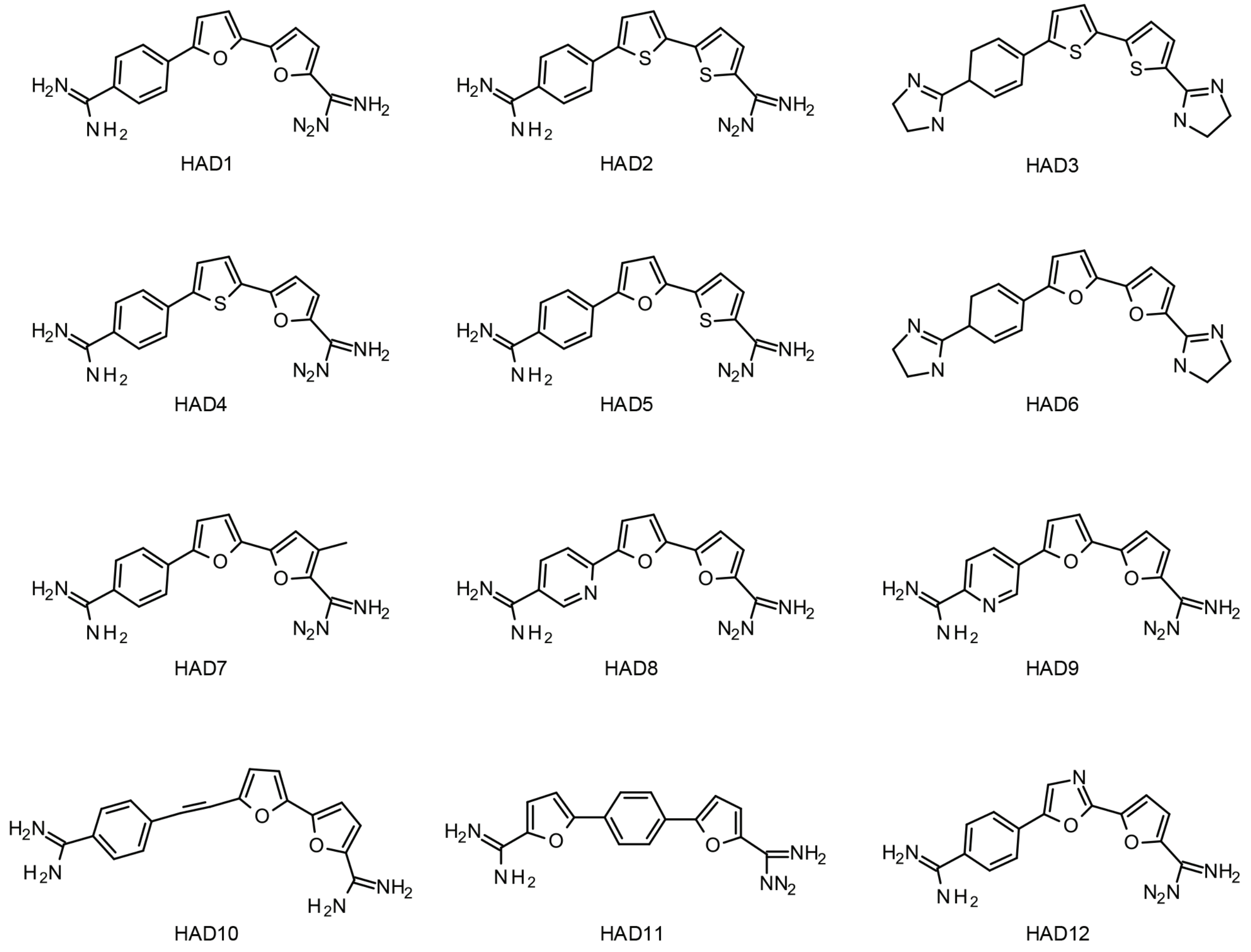

Supplementary Scheme S1: Chemical structures of tested HAD derivatives.

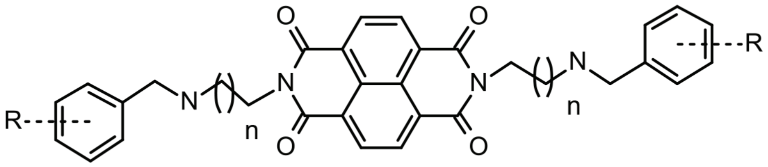

Compound	n	R
NDI1	2	2-OCH_3
NDI2	1	2-OCH_3
NDI3	2	2,3,4-OCH_3
NDI4	2	3,4,5-OCH_3
NDI5	2	H

Supplementary Scheme S2: Chemical structures of tested NDI derivatives.

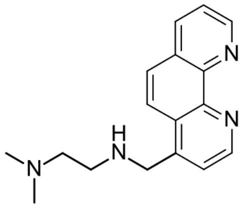

K34

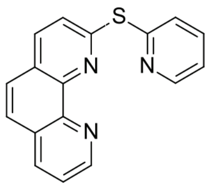

P120

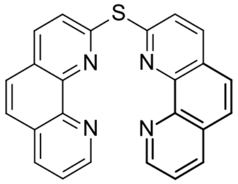

P115

Supplementary Scheme S3: Chemical structures of tested Phen derivatives.

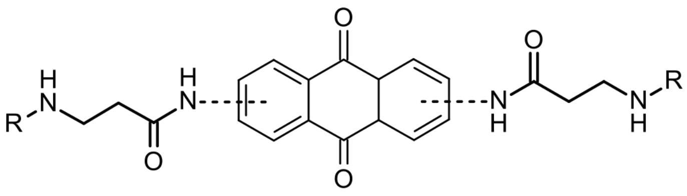

Compound	R	Position
AQ1	Lys	1,5
AQ2	Lys	1,8
AQ3	Lys	2,6
AQ4	Lys	2,7
AQ5	Phe-Lys	1,5
AQ6	Phe-Lys	1,8
AQ7	Phe-Lys	2,6
AQ8	Phe-Lys	2,7

Supplementary Scheme S4: Chemical structures of tested AQ derivatives.

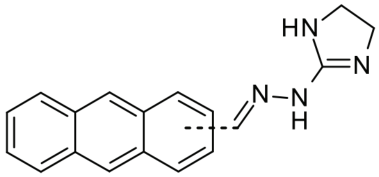

Compound	Position
AN1	1
AN2	2
AN3	9
AN4	9,10
AN5	1,5
AN6	1,8
AN7	2,6
AN8	2,7

Supplementary Scheme S5: Chemical structures of tested AN derivatives.

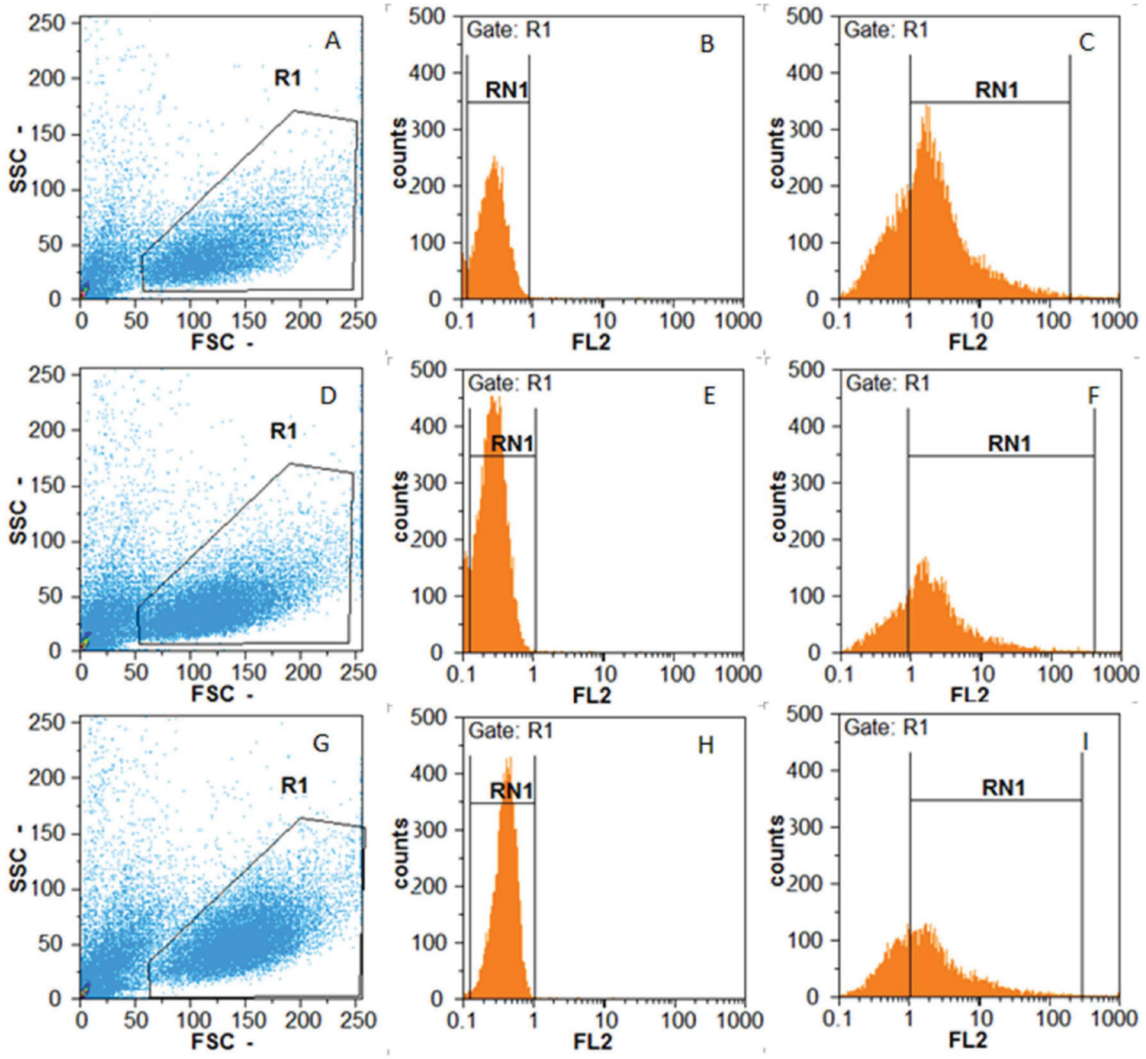

Supplementary Figure S1: Evaluation of CD117 expression in HCG27 cells after 48h of incubation. Control sample: **A.** morphological scatter (forward scatter, FSC; side scatter, SSC); **B.** isotype control, histogram; **C.** CD117 expression, histogram. HCG27 cells incubated with DMSO **D.** morphological scatter (forward scatter, FSC; side scatter, SSC); **E.** isotype control, histogram; **F.** CD117 expression, histogram. HCG27 cells incubated with AQ1 1 μM: **G.** morphological scatter (forward scatter, FSC; side scatter, SSC); **H.** isotype control, histogram **I.** CD117 expression, histogram.

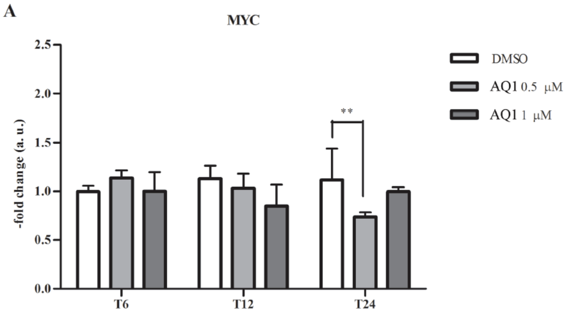

Source of Variation	% of total variation	P value
Interaction	32.11	0.0626
TREATMENT	10.16	0.2076
TIME	4.52	0.4804

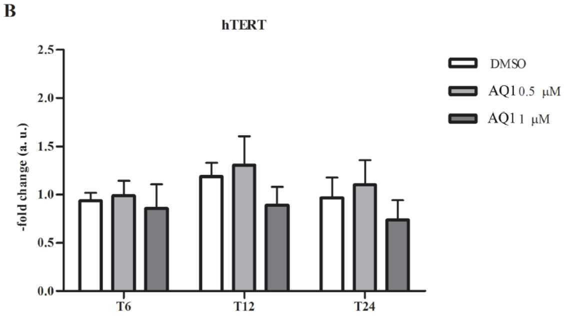

Source of Variation	% of total variation	P value
Interaction	4.77	0.7970
TREATMENT	28.13	0.0203
TIME	15.18	0.0995

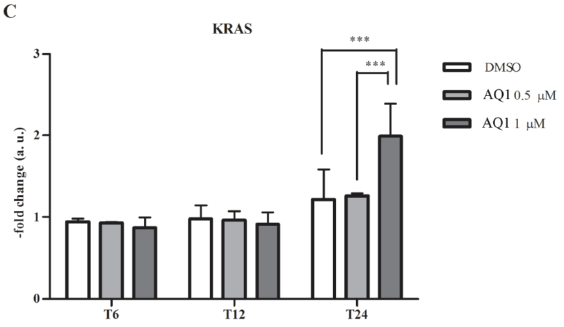

Source of Variation	% of total variation	P value
Interaction	23.49	0.0047
TREATMENT	7.14	0.0596
TIME	49.98	< 0.0001

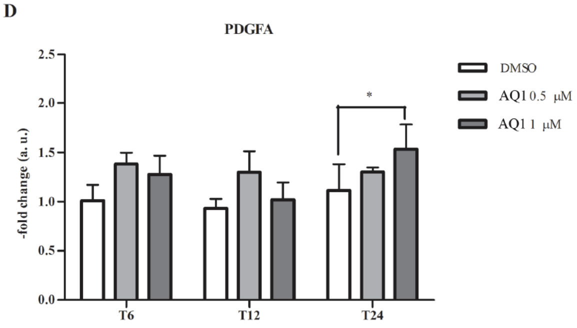

Source of Variation	% of total variation	P value
Interaction	13.82	0.2131
TREATMENT	31.78	0.0044
TIME	15.95	0.0440

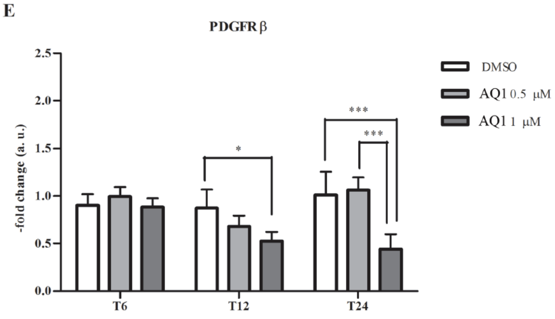

Source of Variation	% of total variation	P value
Interaction	23.27	0.0141
TREATMENT	35.78	0.0003
TIME	16.05	0.0114

Supplementary Figure S2: Effect of AQ1 on mRNA of other oncogenes possessing G4 structures in the HGC27 cell line. **A.** *MYC*, **B.** *hTERT*, **C.** *KRAS*, **D.** *PDGFA* and **E.** *PDGFRβ* mRNA levels were measured using qPCR assays, and data (arithmetic means ± S.D.) are expressed as n-fold change (arbitrary units, a.u.) normalized to the RQ of control cells at each time (T_6, T_{12}, T_{24}), to whom an arbitrary value of 1 was assigned. Two-way ANOVA and Bonferroni post-test were used to identify statistical differences between doses and time of treatment. *,**,***: *P*<0.05; *P*<0.01; *P* <0.001.

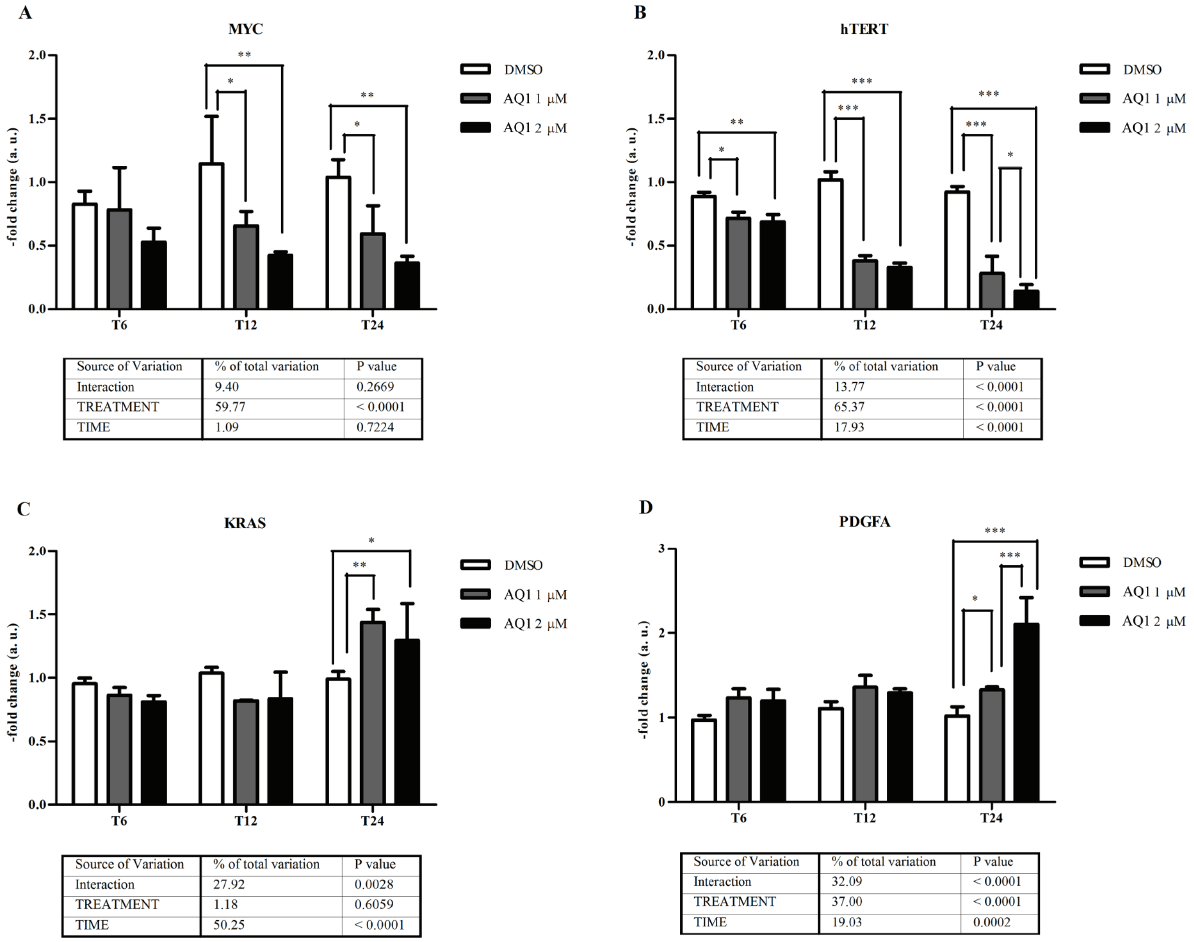

A

Source of Variation	% of total variation	P value
Interaction	9.40	0.2669
TREATMENT	59.77	< 0.0001
TIME	1.09	0.7224

B

Source of Variation	% of total variation	P value
Interaction	13.77	< 0.0001
TREATMENT	65.37	< 0.0001
TIME	17.93	< 0.0001

C

Source of Variation	% of total variation	P value
Interaction	27.92	0.0028
TREATMENT	1.18	0.6059
TIME	50.25	< 0.0001

D

Source of Variation	% of total variation	P value
Interaction	32.09	< 0.0001
TREATMENT	37.00	< 0.0001
TIME	19.03	0.0002

Supplementary Figure S3: Effect of AQ1 on mRNA of oncogenes possessing G4 structures in MCF7 cell line. A. *MYC*, **B.** *hTERT*, **C.** *KRAS* and **D.** *PDGFA* mRNA levels were measured using qPCR assays, and data (arithmetic means ± S.D.) are expressed as n-fold change (arbitrary units, a.u.) normalized to the RQ of control cells at each time (T_6, T_{12}, T_{24}), to whom an arbitrary value of 1 was assigned. Two-way ANOVA and Bonferroni post-test were used to identify statistical differences between doses and time of treatment. $^{*, **, ***}$: $p<0.05$; $p<0.01$; $p<0.001$.

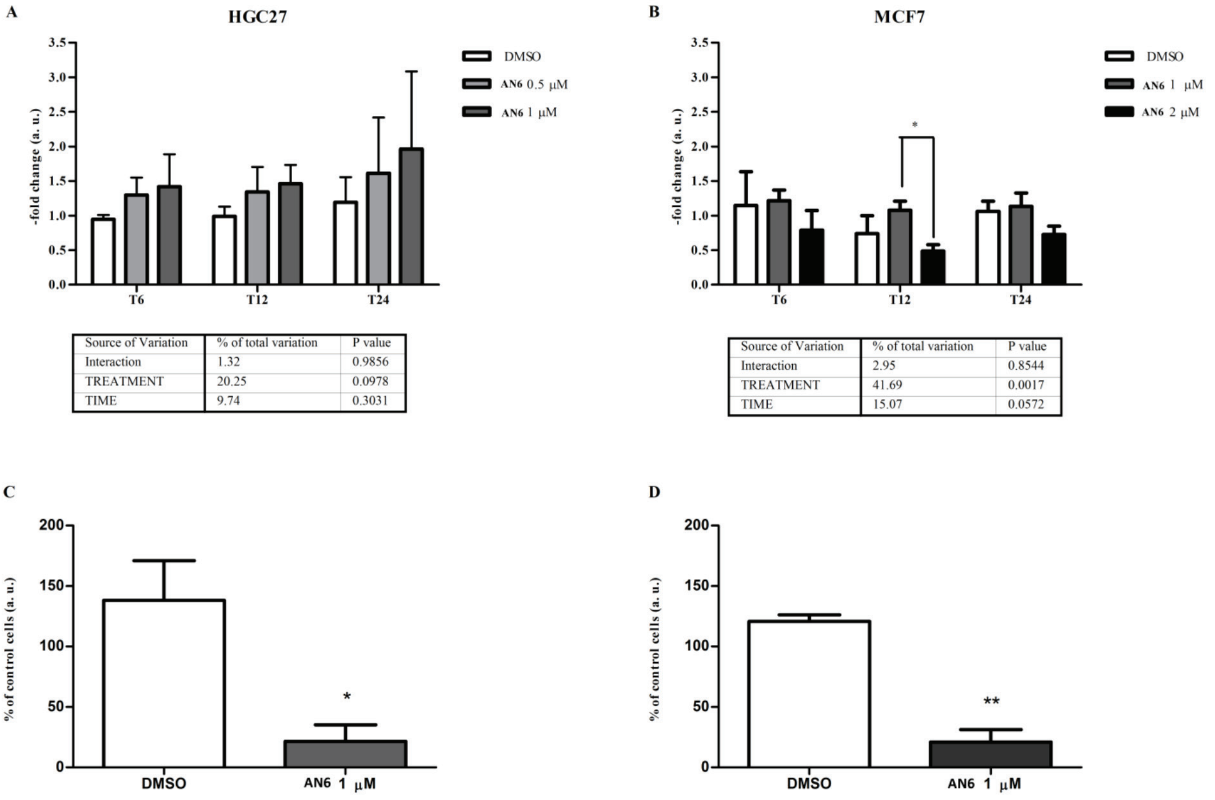

Source of Variation	% of total variation	P value
Interaction	1.32	0.9856
TREATMENT	20.25	0.0978
TIME	9.74	0.3031

Source of Variation	% of total variation	P value
Interaction	2.95	0.8544
TREATMENT	41.69	0.0017
TIME	15.07	0.0572

Supplementary Figure S4: Effect of AN6 on *c-KIT* mRNA and protein expression in HGC27 and MCF7 cell lines. *c-KIT* mRNA levels **A.** and **B.** were measured using a qPCR approach, and data (arithmetic means ± S.D.) are expressed as n-fold change (a. u.) normalized to the RQ value of control cells at each time (T_6, T_{12}, T_{24}), to whom an arbitrary value of 1 was assigned. The two-way ANOVA followed by Bonferroni post-test were used to identify statistical differences between ligand concentrations and time of treatment. The c-kit protein amount **C.** and **D.** was measured by flow cytometry, and data are expressed as n-fold change (%) of the mean fluorescence intensity (MFI) of untreated cells. The Student t-test was used to identify statistical differences between cells exposed to AN6 and those incubated with the vehicle (DMSO).[*,**]: P<0.05; P<0.01.

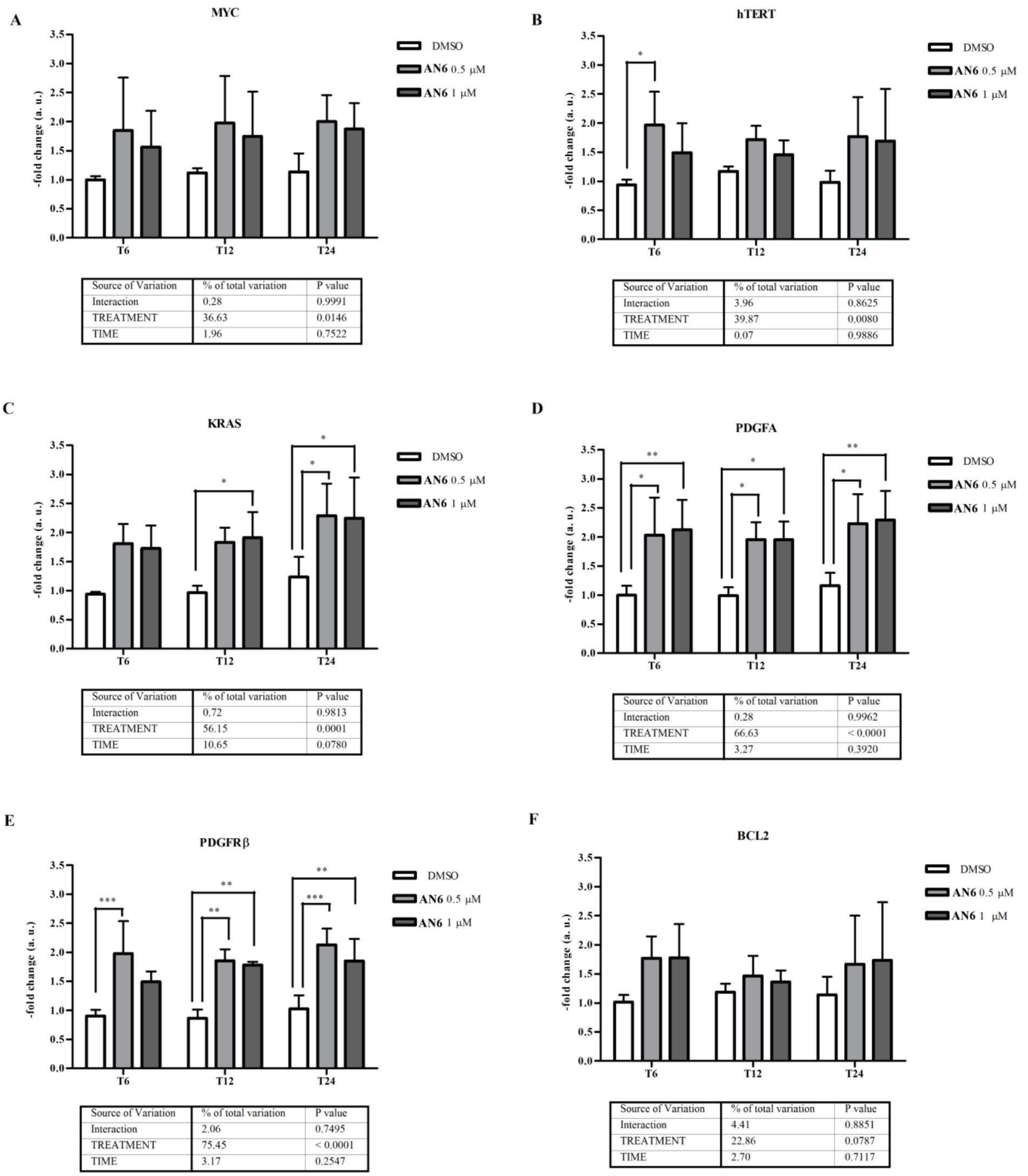

A. MYC

Source of Variation	% of total variation	P value
Interaction	0.28	0.9991
TREATMENT	36.63	0.0146
TIME	1.96	0.7522

B. hTERT

Source of Variation	% of total variation	P value
Interaction	3.96	0.8625
TREATMENT	39.87	0.0080
TIME	0.07	0.9886

C. KRAS

Source of Variation	% of total variation	P value
Interaction	0.72	0.9813
TREATMENT	56.15	0.0001
TIME	10.65	0.0780

D. PDGFA

Source of Variation	% of total variation	P value
Interaction	0.28	0.9962
TREATMENT	66.63	< 0.0001
TIME	3.27	0.3920

E. PDGFRβ

Source of Variation	% of total variation	P value
Interaction	2.06	0.7495
TREATMENT	75.45	< 0.0001
TIME	3.17	0.2547

F. BCL2

Source of Variation	% of total variation	P value
Interaction	4.41	0.8851
TREATMENT	22.86	0.0787
TIME	2.70	0.7117

Supplementary Figure S5: Effect of treatment with AN6 on mRNA of oncogenes possessing G4 structures in the HGC27 cell line. A. *MYC*, **B.** *hTERT*, **C.** *KRAS*, **D.** *PDGFA*, **E.** *PDGFRβ*, and **F.** *BCL2* mRNA levels were measured using qPCR assays, and data (arithmetic means ± S.D.) are expressed as n-fold change (a.u.) normalized to the RQ of control cells at each time (T_6, T_{12}, T_{24}) to which an arbitrary value of 1 was assigned. Two-way ANOVA and Bonferroni post-test were used to assess statistical differences between doses and time of treatment. $^{*, **, ***}$: p<0.05; p<0.01; p<0.001.

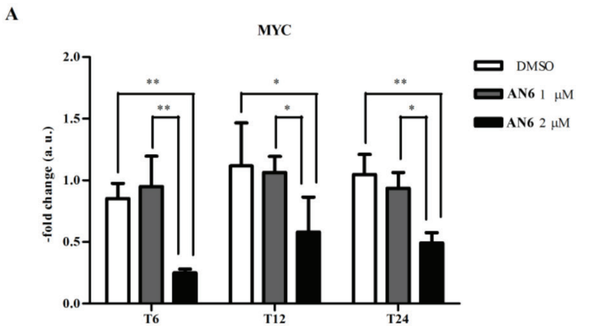

Source of Variation	% of total variation	P value
Interaction	2.24	0.7943
TREATMENT	64.71	< 0.0001
TIME	8.87	0.0600

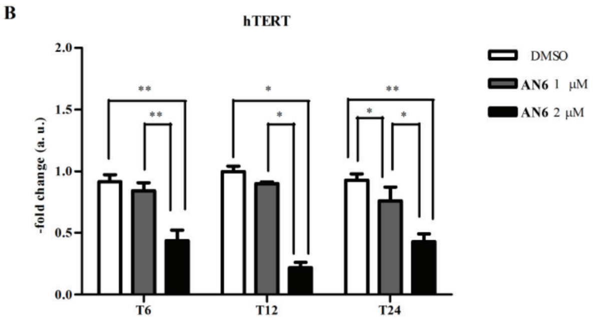

Source of Variation	% of total variation	P value
Interaction	6.69	0.0009
TREATMENT	89.17	< 0.0001
TIME	0.20	0.6344

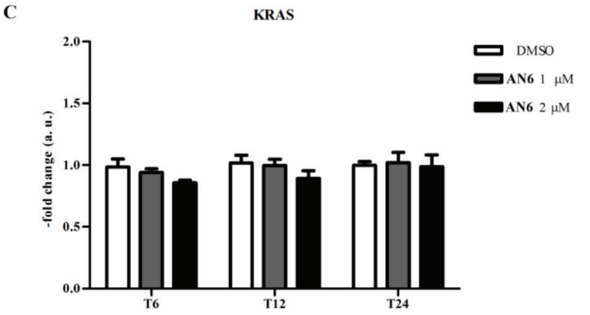

Source of Variation	% of total variation	P value
Interaction	9.36	0.4725
TREATMENT	28.05	0.0134
TIME	16.93	0.0585

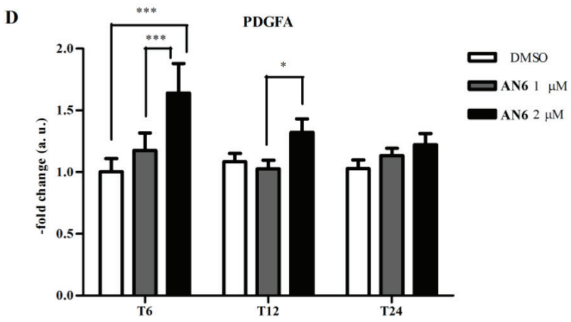

Source of Variation	% of total variation	P value
Interaction	17.94	0.0191
TREATMENT	51.93	< 0.0001
TIME	9.36	0.0352

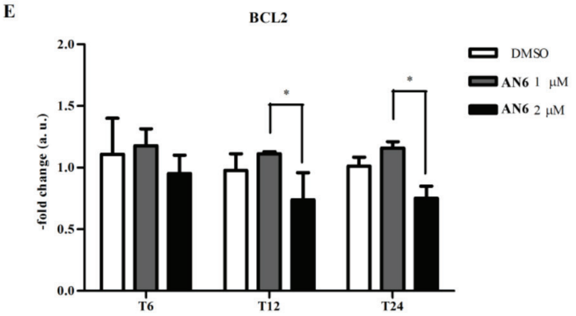

Source of Variation	% of total variation	P value
Interaction	2.62	0.8760
TREATMENT	48.97	0.0007
TIME	8.72	0.1675

Supplementary Figure S6: Effect of exposure with AN6 on mRNA of oncogenes possessing G4 structures in the MCF7 cell line. A. *MYC*, **B.** *hTERT*, **C.** KRAS, **D.** *PDGFA* and **E.** *BCL2* mRNA level were measured by using a qPCR assay, and data (arithmetic means ± S.D.) are expressed as n-fold change (a. u.) normalized to the RQ of control cells at each time (T_6, T_{12}, T_{24}) to which an arbitrary value of 1 was assigned. Two-way ANOVA followed by Bonferroni post-test was used to assess statistical differences between doses and time of treatment. $^{*, **, ***}$: p<0.05; p<0.01; p<0.001.

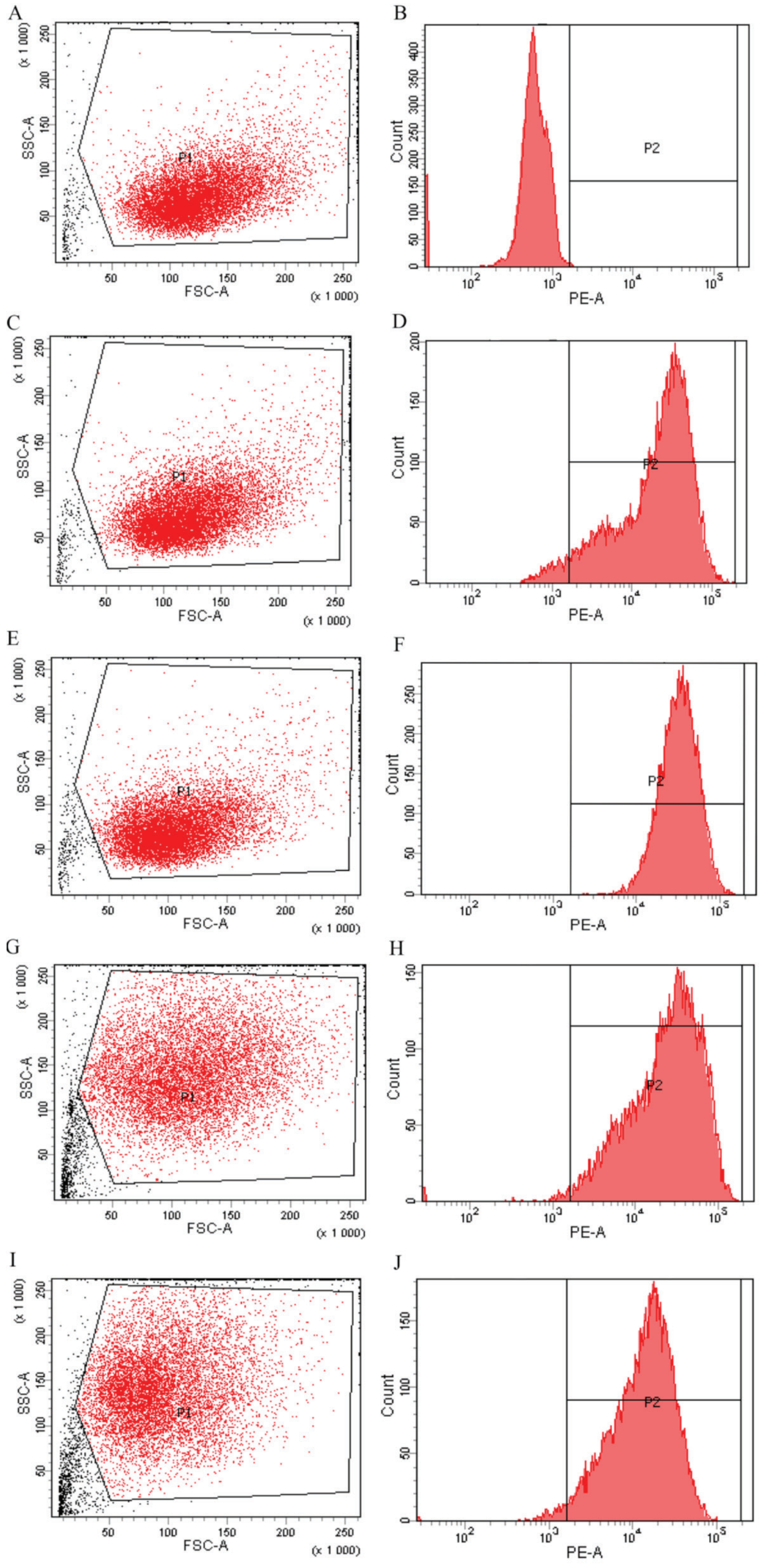

Supplementary Figure S7: Flow cytometry analysis of CD117 in the α155 cell line. Morphological scatter plot with forward scatter (FSC) vs side scatter (SSC) and c-kit histogram plot of fluorescence intensity (FI) of different samples: **A.** and **B.** irrelevant antibody IgG; **C.** and **D.** control cells; **E.** and **F.** DMSO treated cells FI; **G.** and **H.** AQ1 1 µM treated cells; **I.** and **J.** AQ1 2 µM treated cells.

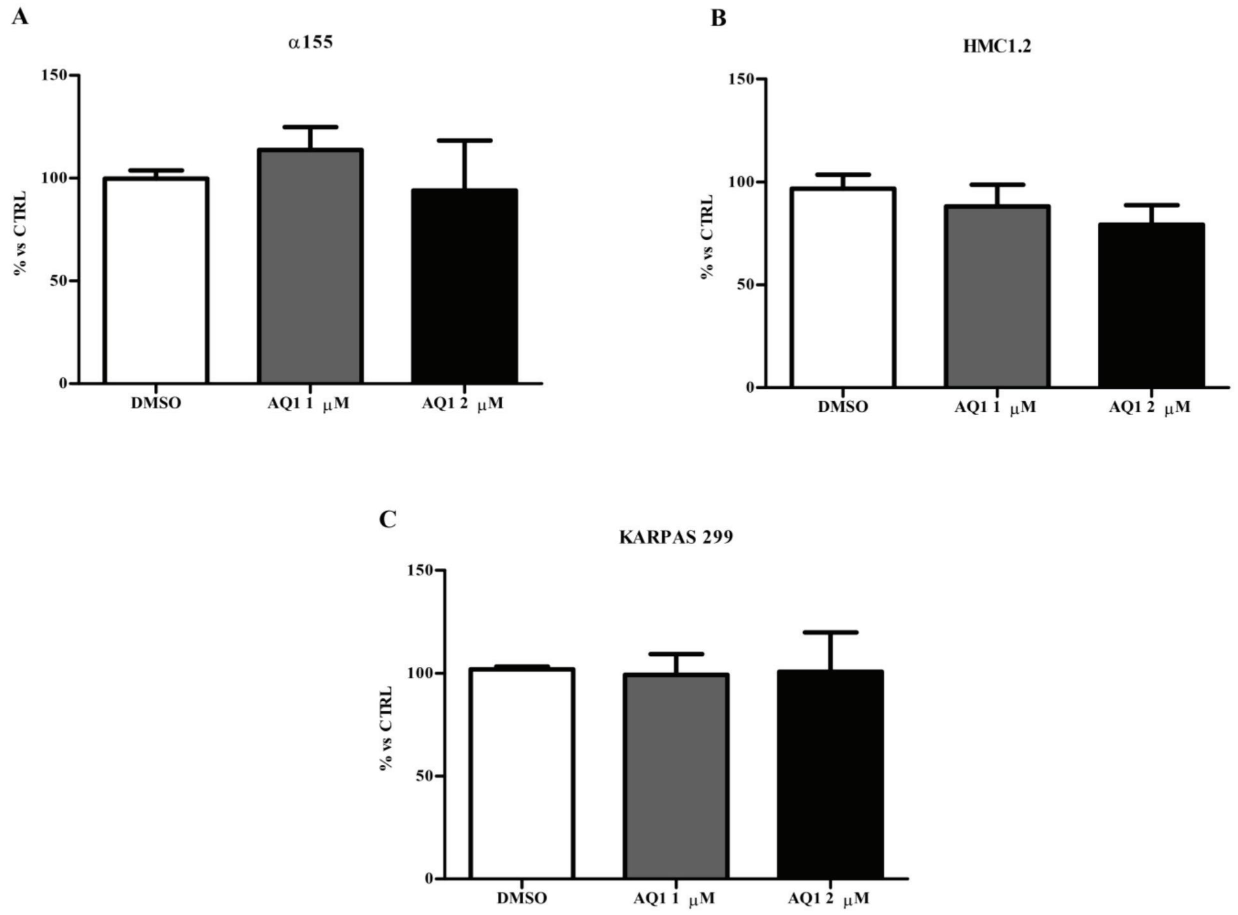

Supplementary Figure S8: Effect of treatment with AQ1 on HLA proteins of α155 A. HMC1.2 B. and KARPAS299 C. The HLA protein amounts were measured by flow cytometry and data are expressed as percentage of the fluorescence intensity (FI) measured in untreated cells (control). One-way ANOVA with Bonferroni post-test were used to assess statistical differences between cell treated with AQ1 and those treated with the vehicle (DMSO).

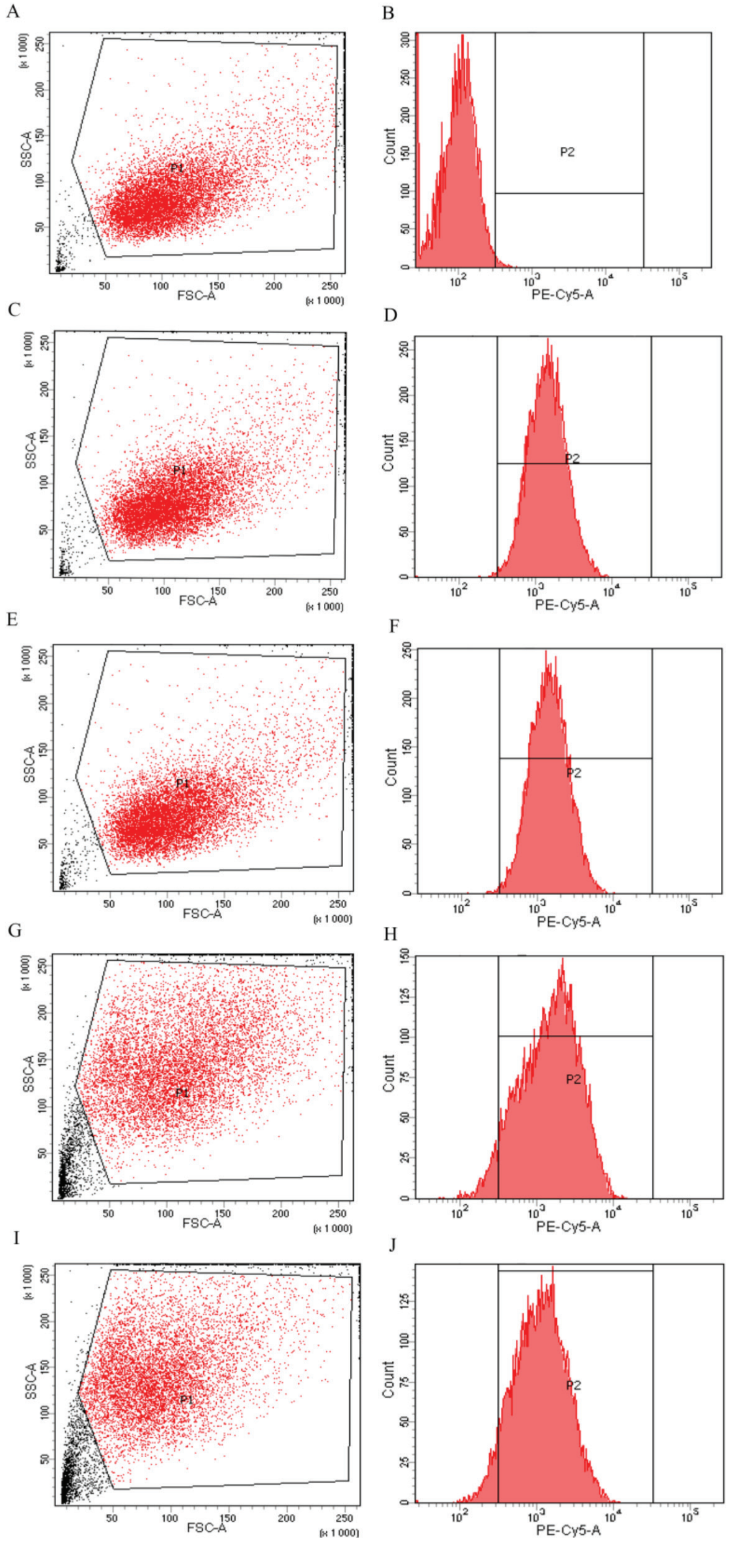

Supplementary Figure S9: Flow cytometry analysis of HLA in α155 cell line. Morphological scatter plot with forward scatter (FSC) vs side scatter (SSC) and HLA histogram plot of fluorescence intensity (FI) of different samples. **A.** and **B.** irrelevant antibody IgG; **C.** and **D.** control cells; **E.** and **F.** DMSO treated cells FI; **G.** and **H.** AQ1 1 μM treated cells; **I.** and **J.** AQ1 2 μM treated cells.

Supplementary Table S1: Library of tested compounds and variation of the melting temperature they induced at 1 µM concentration of each tested DNA sequence

Compounds	Ref.	Kit1	Kit2	HTS	dsDNA
HAD1	DB832[a]	12.5	13.3	10.9	2.4
HAD2	DB1450[a]	21.8	11.3	12.8	12.1
HAD3	DB2037[a]	19.6	17.5	16.8	7.8
HAD4	DB1463[a]	10.1	1.2	7.3	2.0
HAD5	DB1438[a]	3.5	11.0	13.3	1.2
HAD6	DB1972[a]	14.0	13.1	9.9	4.2
HAD7	DB1949[a]	15.7	13.4	12.6	2.6
HAD8	DB934[a]	7.4	5.8	5.0	2.9
HAD9	DB1693[a]	12.1	9.5	9.4	2.2
HAD10	DB1694[a]	10.6	13.9	5.6	1.1
HAD11	DB1093[a]	12.9	13.4	11.7	11.5
HAD12	DB1999[a]	8.1	13.1	4.9	2.4
NDI1	2[b]	1.5	8.3	10.3	3.0
NDI2	1[b]	0.6	0.7	9.2	2.8
NDI3	20[b]	2.0	3.3	13.5	4.4
NDI4	22[b]	0.4	0.7	7.0	1.9
NDI5	8[b]	0.2	0.5	10.1	5.0
Phen1	K34[c]	0.0	0.0	0.1	0.0
Phen1_Ni(II)	$(K34)_2Ni(II)$[c]	1.9	0.0	3.1	0.0
Phen2	P120[d]	0.0	0.0	0.1	0.0
Phen2_Ni(II)	$(P120)_2Ni(II)$[d]	1.8	0.0	10.0	0.0
Phen3	P115[e]	2.5	5.2	0.1	0.0
Phen3_Ni(II)	(P115)Ni(II)[e]	30.9	30.6	23.6	0.3
AQ1	D-13[f]	13.1	15.3	18.0	4.6
AQ2	E-13[f]	6.7	8.4	18.9	4.5
AQ3	B-13[f]	9.9	12.9	14.2	2.5
AQ4	C-13[f]	5.2	5.1	9.9	1.4
AQ5	D-15[f]	10.2	6.0	18.2	1.2
AQ6	E-15[f]	13.0	1.7	4.5	0.1
AQ7	B-15[f]	9.5	11.1	7.0	0.3
AQ8	C-15[f]	4.0	6.4	4.3	0.1
AN1	Ant1[g]	0.0	2.4	1.0	1.2
AN2	Ant2[g]	0.0	0.1	0.3	0.6
AN3	Ant9[g]	0.0	0.2	1.0	1.6
AN4	Ant9,10[g]	5.0	6.5	1.7	0.9

(Continued)

Compounds	Ref.	Kit1	Kit2	HTS	dsDNA
AN5	Ant1,5[g]	2.0	6.7	13.6	0.5
AN6	Ant1,8[g]	5.2	8.0	3.0	0.8
AN7	Ant2,6[g]	4.4	7.2	4.7	0.1
AN8	Ant2,7[g]	1.2	3.6	2.0	0.1

Errors were ± 0.4 °C. The compound name previously used and the corresponding reference are reported in the ref column.
[a]: Nanjunda R, Musetti C, Kumar A, Ismail MA, Farahat AA, Wang S, Sissi C, Palumbo M, Boykin DW, Wilson WD. Heterocyclic dications as a new class of telomeric G-quadruplex targeting agents. Curr Pharm Des. 2012; 18: 1934-1947.
[b]: Milelli A, Tumiatti V, Micco M, Rosini M, Zuccari G, Raffaghello L, Bianchi G, Pistoia V, Díaz JF, Pera B, Trigili C, Barasoain I, Musetti C, et al. Structure-activity relationships of novel substituted naphthalene diimides as anticancer agents. Eur J Med Chem. 2012; 57: 417-428.

[c]: Musetti C, Lucatello L, Bianco S, Krapcho AP, Cadamuro SA, Palumbo M, Sissi C. Metal ion-mediated assembly of effective phenanthroline-based G-quadruplex ligands. Dalton Trans. 2009; 21: 3657-3660.

[d]: Bianco S, Musetti C, Krapcho AP, Palumbo M, Sissi C. Ni2+ and Cu2+ complexes of a phenanthroline-based ligand bind to G-quadruplexes at non-overlapping sites. Chem Commun (Camb). 2013; 49: 8057-8059.

[e]: Bianco S, Musetti C, Waldeck A, Sparapani S, Seitz JD, Krapcho AP, Palumbo M, Sissi C. Bis-phenanthroline derivatives as suitable scaffolds for effective G-quadruplex recognition. Dalton Trans. 2010; 39: 5833-5841.

[f]: Zagotto G, Ricci A, Vasquez E, Sandoli A, Benedetti S, Palumbo M, Sissi C. Tuning G-quadruplex vs double-stranded DNA recognition in regioisomeric lysyl-peptidyl-anthraquinone conjugates. Bioconjug Chem. 2011; 22: 2126-2135.

[g]: Folini M, Pivetta C, Zagotto G, De Marco C, Palumbo M, Zaffaroni N, Sissi C. Remarkable interference with telomeric function by a G-quadruplex selective bisantrene regioisomer. Biochem Pharmacol. 2010; 70: 1781-1790.

Supplementary Table S2: qPCR and flow cytometry experimental settings chosen for each cell line and ligand tested in the study

Cell line	Ligand	IC_{50} (µM)	Ligand concentrations for qPCR assay (µM)	Analyzed exposure times (qPCR)	Ligand concentrations for flow cytometry assay (µM)	Analyzed exposure times (flow cytometry)
HCG27	AQ1	1.65	0.5 – 1.0	T6, T12, T24	1.0 – 2.0[a]	T48
	AQ7	> 10.0	10.0[b]	T6, T12, T24	--	--
	AN6	2.04	0.5 – 1.0	T6, T12, T24	1.0	T48
MCF7	AQ1	3.0	1.0 – 2.0	T6, T12, T24	1.0 – 2.0[a]	T48
	AQ7	> 10.0	10.0[b]	T6, T12, T24	--	--
	AN6	2.70	1.0 – 2.0	T6, T12, T24	1.0	T48
α155	AQ1	--	1.0	T6, T12	1.0 – 2.0	T48
HMC1.2	AQ1	--	1.0	T6, T12	1.0 – 2.0	T48

[a] 2 µM concentration was used only in BCL2 detection experiment (Figure 8); [b] data not shown in the manuscript.

Supplementary Table S3: Primers and probes used for the qPCR analysis either obtained from previous publications or specifically designed for this study

Gene	UPL[a] probe	Primers (5'- 3')	Source
MYC [b]	#67	F [l]: TGGTGCTCCATGAGGAGACA<br>R [m]: GTGGCACCTCTTGAGGACCA	Gunaratnam et al., 2009
PDGFA [c]	#77	F: ACACGAGCAGTGTCAAGTGC<br>R: CCTGCAGTATTCCACCTTGG	Iqbal et al., 2012
PDGFRB [d]	#14	R: TGCTCATCTGTGAAGGCAAG<br>F: TGGCATTGTAGAACTGCTCG	Chanakira et al., 2012
BCL2 [e]	#75	F: ATGTGTGTGGAGAGCGTCAA<br>R: GCCGTACAGTTCCACAAAGG	Brassesco et al., 2010
B2M [f]	#42	F: AGGCTATCCAGCGTACTCCA<br>R: TGTCGGATGGATGAAACCCA	designed *ex novo*
GAPDH [g]	#60	F: CTCTGCTCCTCCTGTTCGAC<br>R: ACGACCAAATCCGTTGACTC	designed *ex novo*
HPRT1 [h]	#22	F: TGATAGATCCATTCCTATGACTGTAGA<br>R: CAAGACATTCTTTCCAGTTAAAGTTG	designed *ex novo*
KIT [i]	#29	F: GGCACGGTTGAATGTAAGGC<br>R: CAGGGTGTGGGGATGGATTT	designed *ex novo*
KRAS [j]	#62	F: GGAGCTGGTGGCGTAGGCAAG<br>R: GCCCTCCCCAGTCCTCATGT	designed *ex novo*
hTERT [k]	#68	F: GGAGAACAAGCTGTTTGCGG<br>R: AGCCATACTCAGGGACACCT	designed *ex novo*

[a]Universal Probe Library; [b]V-Myc Avian Myelocytomatosis Viral Oncogene Homolog; [c]Platelet-Derived Growth Factor Alpha Polypeptide; [d]*beta*-type platelet-derived growth factor receptor; [e]B-cell lymphoma 2; [f]β-2-Microglobulin; [g]Glyceraldehyde-3-Phosphate Dehydrogenase; [h]Hypoxanthine Phosphoribosyltransferase 1; [i]V-Kit Hardy-Zuckerman 4 Feline Sarcoma Viral Oncogene Homolog; [j]Kirsten rat sarcoma viral oncogene homolog; [k]Telomerase Reverse Transcriptase; [l]forward; [m]reverse.

Supplementary Table S4: qPCR assay standard curve parameters obtained in MCF7 and HGC27 cell lines

Gene	MCF7			HGC27		
	Slope	Efficiency (%)	Dynamic range (Ct)	Slope	Efficiency (%)	Dynamic range (Ct)
B2M	-3,192	105,7	18,78 - 32,06	-3,34	99,1	18,74 - 30,85
BCL2	-3,41	96,5	25,82 - 34,13	-3,27	102,2	24,90 - 33,43
GAPDH	-3,48	93,7	15,81 - 30,59	-3,28	102	16,20 - 30,64
HPRT1	-3,324	99,9	20,23 - 32,63	-3,3	101	20,96 - 33,18
KIT	-3,137	108,3	29,01 - 35,78	-3,531	92	29,58 - 39,58
KRAS	-3,34	99	21,50 - 32,28	3,352	98,8	21,30 - 32,41
MYC	-3.3	100.7	26.85 - 33.06	-3,502	93	22,47 - 30,89
PDGFA	-3,169	106,8	22,97 - 31,68	-3,18	106,3	23,87 - 33,02
PDGFRB		Not expressed		-3,36	98,4	24,84 - 32,28
hTERT	-3,11		33,24 - 38,82	-3,34	99,2	29,38 - 35,78

Supplementary Table S5: qPCR assay standard curve parameters obtained in α155 and HMC1.2 cell lines

Gene	α155			HMC1.2		
	Slope	Efficiency (%)	Dynamic range (Ct)	Slope	Efficiency (%)	Dynamic range (Ct)
B2M	-3.33	99.6	17.18-31.68	-3.38	97.7	17.46-31.25
GAPDH	-3.37	98	16.43-30.91	-3.40	96.7	17.34-31.36
HPRT1	-3.39	97.1	21.75-35.65	-3.38	97.5	22.14-37.67
KIT	-3.32	99.7	18.00-32.32	-3.30	101	19.08-32.90
